# Duckweed Evolution: from Land back to Water

**DOI:** 10.1093/gpbjnl/qzaf074

**Published:** 2025-08-23

**Authors:** Yang Fang, Xueping Tian, Yanling Jin, Anping Du, Yanqiang Ding, Zhihua Liao, Kaize He, Yonggui Zhao, Ling Guo, Yao Xiao, Yaliang Xu, Shuang Chen, Yuqing Che, Li Tan, Songhu Wang, Jiatang Li, Zhuolin Yi, Lanchai Chen, Leyi Zhao, Fangyuan Zhang, Guoyou Li, Jinmeng Li, Qinli Xiong, Yongmei Zhang, Qing Zhang, Xuan Hieu Cao, Hai Zhao

**Affiliations:** Agricultural Microbial Agents Key Laboratory of Sichuan Province, National Engineering and Research Center for Natural Medicines, Chengdu Institute of Biology, Chinese Academy of Sciences, Chengdu 610213, China; Agricultural Microbial Agents Key Laboratory of Sichuan Province, National Engineering and Research Center for Natural Medicines, Chengdu Institute of Biology, Chinese Academy of Sciences, Chengdu 610213, China; Agricultural Microbial Agents Key Laboratory of Sichuan Province, National Engineering and Research Center for Natural Medicines, Chengdu Institute of Biology, Chinese Academy of Sciences, Chengdu 610213, China; Agricultural Microbial Agents Key Laboratory of Sichuan Province, National Engineering and Research Center for Natural Medicines, Chengdu Institute of Biology, Chinese Academy of Sciences, Chengdu 610213, China; Agricultural Microbial Agents Key Laboratory of Sichuan Province, National Engineering and Research Center for Natural Medicines, Chengdu Institute of Biology, Chinese Academy of Sciences, Chengdu 610213, China; Institute of Digestive Disease and Department of Medicine and Therapeutics, State Key Laboratory of Digestive Disease, Li Ka Shing Institute of Health Sciences, CUHK–Shenzhen Research Institute, The Chinese University of Hong Kong, Hong Kong Special Administrative Region 999077, China; Key Laboratory of Eco-environments in Three Gorges Reservoir Region (Ministry of Education), SWU-TAAHC Medicinal Plant Joint R&D Center, School of Life Sciences, Southwest University, Chongqing 400715, China; Agricultural Microbial Agents Key Laboratory of Sichuan Province, National Engineering and Research Center for Natural Medicines, Chengdu Institute of Biology, Chinese Academy of Sciences, Chengdu 610213, China; School of Ecology and Environmental Sciences & Yunnan Key Laboratory for Plateau Mountain Ecology and Restoration of Degraded Environments, Yunnan University, Kunming 650091, China; Agricultural Microbial Agents Key Laboratory of Sichuan Province, National Engineering and Research Center for Natural Medicines, Chengdu Institute of Biology, Chinese Academy of Sciences, Chengdu 610213, China; Department of Pediatrics, Children Hematological Oncology and Birth Defects Laboratory, The Affiliated Hospital of Southwest Medical University, Sichuan Clinical Research Center for Birth Defects, Luzhou 646000, China; Analytical and Testing Center, Sichuan University of Science and Engineering, Zigong 643000, China; Agricultural Microbial Agents Key Laboratory of Sichuan Province, National Engineering and Research Center for Natural Medicines, Chengdu Institute of Biology, Chinese Academy of Sciences, Chengdu 610213, China; Institute of Urban Agriculture, Chinese Academy of Agricultural Sciences, Chengdu 610213, China; Agricultural Microbial Agents Key Laboratory of Sichuan Province, National Engineering and Research Center for Natural Medicines, Chengdu Institute of Biology, Chinese Academy of Sciences, Chengdu 610213, China; Agricultural Microbial Agents Key Laboratory of Sichuan Province, National Engineering and Research Center for Natural Medicines, Chengdu Institute of Biology, Chinese Academy of Sciences, Chengdu 610213, China; Institute of Crop Sciences, Chinese Academy of Agricultural Sciences, Beijing 100081, China; Agricultural Microbial Agents Key Laboratory of Sichuan Province, National Engineering and Research Center for Natural Medicines, Chengdu Institute of Biology, Chinese Academy of Sciences, Chengdu 610213, China; Agricultural Microbial Agents Key Laboratory of Sichuan Province, National Engineering and Research Center for Natural Medicines, Chengdu Institute of Biology, Chinese Academy of Sciences, Chengdu 610213, China; Agricultural Microbial Agents Key Laboratory of Sichuan Province, National Engineering and Research Center for Natural Medicines, Chengdu Institute of Biology, Chinese Academy of Sciences, Chengdu 610213, China; Agricultural Microbial Agents Key Laboratory of Sichuan Province, National Engineering and Research Center for Natural Medicines, Chengdu Institute of Biology, Chinese Academy of Sciences, Chengdu 610213, China; Agricultural Microbial Agents Key Laboratory of Sichuan Province, National Engineering and Research Center for Natural Medicines, Chengdu Institute of Biology, Chinese Academy of Sciences, Chengdu 610213, China; School of Food and Bioengineering, Xihua University, Chengdu 610039, China; Pitzer college, California, CA 91711, USA; Key Laboratory of Eco-environments in Three Gorges Reservoir Region (Ministry of Education), SWU-TAAHC Medicinal Plant Joint R&D Center, School of Life Sciences, Southwest University, Chongqing 400715, China; Agricultural Microbial Agents Key Laboratory of Sichuan Province, National Engineering and Research Center for Natural Medicines, Chengdu Institute of Biology, Chinese Academy of Sciences, Chengdu 610213, China; Agricultural Microbial Agents Key Laboratory of Sichuan Province, National Engineering and Research Center for Natural Medicines, Chengdu Institute of Biology, Chinese Academy of Sciences, Chengdu 610213, China; Institute of Remote Sensing and Digital Agriculture, Sichuan Academy of Agricultural Sciences, Chengdu 610066, China; Agricultural Microbial Agents Key Laboratory of Sichuan Province, National Engineering and Research Center for Natural Medicines, Chengdu Institute of Biology, Chinese Academy of Sciences, Chengdu 610213, China; Agricultural Microbial Agents Key Laboratory of Sichuan Province, National Engineering and Research Center for Natural Medicines, Chengdu Institute of Biology, Chinese Academy of Sciences, Chengdu 610213, China; School of Food and Bioengineering, Xihua University, Chengdu 610039, China; Department of Plant Physiology, Institute of Biology, Martin Luther University Halle-Wittenberg, 06120 Halle (Saale), Germany; Leibniz Institute of Plant Genetics and Crop Plant Research (IPK), 06466 Seeland, Germany; Forest Genetics and Forest Tree Breeding, University of Goettingen, 37077 Goettingen, Germany; Agricultural Microbial Agents Key Laboratory of Sichuan Province, National Engineering and Research Center for Natural Medicines, Chengdu Institute of Biology, Chinese Academy of Sciences, Chengdu 610213, China

**Keywords:** Aquatic, Duckweed, Evolution, Genome, Terrestrial

## Abstract

Terrestrialization is an important evolutionary process that plants experienced. However, little is known about how land plants acquired aquatic growth behaviors. Here, we integrate multiproxy evidence to elucidate the evolution of the aquatic plant duckweed. Three genera of duckweeds show chronologically gradual degeneration in root structure and stomatal function and a decrease in lignocellulose content, accompanied by the contraction of relevant gene families and/or a decline in their transcription levels. The number of genes in main phytohormone pathways is also gradually decreased. The coordinated action of genes involved in auxin signaling and rhizoid development causes a gradual decrease in adventitious roots. Additionally, the significant expansion of the flavonoid pathway is related to the adaptation of duckweeds to floating growth. This study reconstructs the evolutionary history of duckweeds, tracing its journey from land back to water — a reverse trajectory of early land plants.

## Graphical abstract

**Figure:**
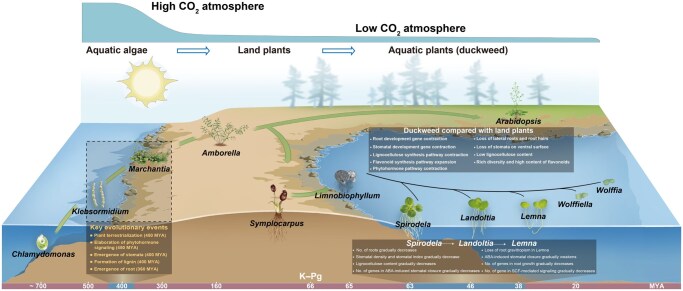

## Introduction

Life on Earth originated in water 4.0–3.8 billion years ago, and aquatic algae first transitioned from water to land 450 million years ago (MYA) [1]. Terrestrialization was first initiated by plants and then followed by animals. During this process, four key traits related to land adaptability emerged in plants around 400 MYA, including stomata [2], lignin [3], roots [4], and more diverse phytohormones [5]. The occurrence of such new characteristics brought about tremendous changes in plant life forms and expanded biodiversity on Earth. In fact, the number of species and total biomass of terrestrial organisms far exceed that of aquatic ones. Although land only accounts for 29% of the total Earth’s surface, terrestrial angiosperms alone contain around 400,000 species — more than 4 times the number of aquatic plant species [6]. Meanwhile, land plants account for 82% of the total biomass in the biosphere [7].

These evolutionary phenomena from water to land have always intrigued scientists in related fields [5]. However, the reverse evolution from land back to water has not been extensively studied at a molecular level. Even though there were studies confirming this process, most of the evidence was derived only from molecular phylogenetic trees established with some genes of aquatic plants [8–10]. These studies lacked evidence on how the key characteristics, functions, and genes of these plants adapted to water.

**Figure 1:**
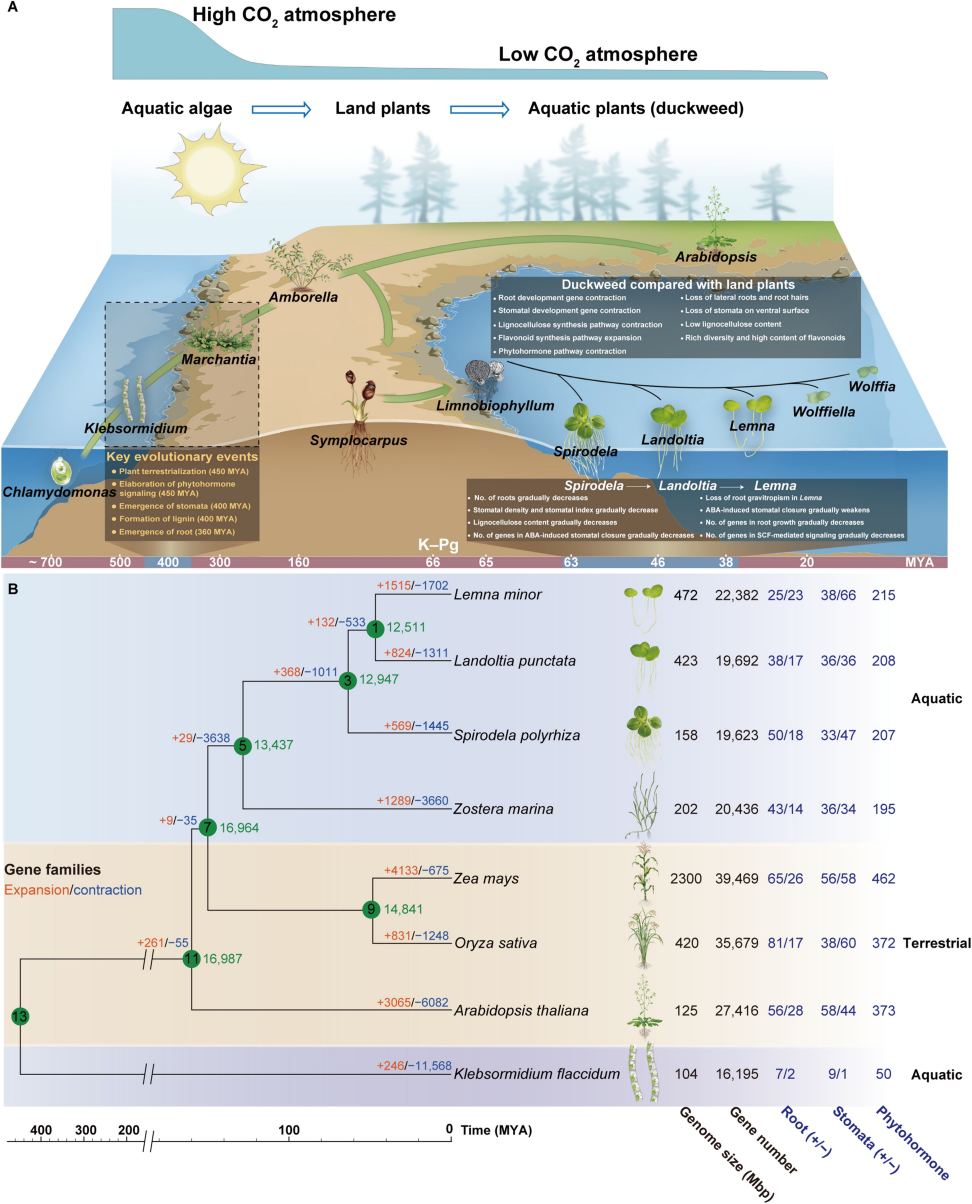
The evolution model, phylogenetic tree, and gene family analysis of duckweeds **A**. Evolution of duckweeds from land back to water. **B**. Phylogenetic tree and gene family analysis of duckweeds. The tree includes five representative species from the Viridiplantae for comparison. The copy number of genes involved in root development (Root), stomatal development (Stomata), and phytohormone pathways (Phytohormone) are shown. “+” and “−” indicate positively and negatively related to development, respectively. The numbers at the nodes of the phylogenetic tree indicate the divergence time. MYA, million years ago; K–Pg, Cretaceous–Paleogene.

Duckweeds may be the answer to reverse terrestrialization. These floating monocots, which are the smallest flowering plants on Earth and belong to the family Lemnaceae, comprise five genera: *Spirodela*, *Landoltia*, *Lemna*, *Wolffiella*, and *Wolffia* [11]. Evolutionarily, *Spirodela* was the most ancient genus among extant duckweeds, followed by *Landoltia*, *Lemna*, *Wolffiella*, and *Wolffia* (**Figure 1**A). Over time, there was a trend of organism size reduction and root loss, consistent with reverse terrestrialization. This pattern is quite different from adaptive changes observed in seagrasses, which are submerged plants living in the sea and have adapted to salinity, light, and CO_2_ availability rather than undergoing simplification in size and root structure [8–10].

Here, we selected *Spirodela polyrhiza* strain 7498, *Landoltia punctata* strain 0202, and *Lemna minor* strain 7753 as representatives of their respective genera and integrated their genomic, transcriptomic, morphological, and physiological data. Our results indicate that the four key terrestrial traits (roots, stomata, phytohormones, and lignocellulose) are degenerating or even lost at multiple levels, including genome, transcription, function, structure, and composition. The study of extant duckweeds and fossils of *Limnobiophyllum scutatum* [12,13] reconstructs the evolutionay process of duckweeds from land back to water.

## Results and discussion

### Duckweed phylogeny

We sequenced, assembled, and annotated the genome of *L. punctata* strain 0202 (GenBank: PRJNA546087; File S1) and performed comparative genomic analysis with two other published duckweed genomes, *S. polyrhiza* strain 7498 and *L. minor* strain 7753, as well as genomes of several model species and evolutionary node species including *Klebsormidium flaccidum*, *Zostera marina*, *Arabidopsis thaliana*, *Oryza sativa*, and *Zea mays* (Table S1). A phylogenetic tree was constructed using genes of 1545 single-copy families shared by these eight species to determine their evolutionary relationships. Our results were consistent with previous studies on the phylogeny and systematics of Lemnaceae [11,14,15]. Meanwhile, we performed comparative transcriptomic analyses among *S. polyrhiza*, *L. punctata*, and *L. minor* to further understand their functional and structural characteristics (Tables S2–S21).

Compared with the alga *K. flaccidum* that contains some land-related genes, land plants (*Z. mays*, *A. thaliana*, and *O. sativa*) harbor a higher number of genes relevant to terrestrial traits such as roots, stomata, and phytohormones. On the contrary, the total number of genes related to these traits in aquatic duckweeds (*S. polyrhiza*, *L. punctata*, and *L. minor*) is greatly reduced compared to typical land plants (Figure 1B). As aquatic angiosperms belonging to the order Alismatales and closely related to the family Araceae, duckweeds diverged early from the basal lineage of Alismatales (*Tofieldia thibetica*) at ∼ 120.0 MYA and from the basal lineage of Araceae (*Symplocarpus renifolius*) at ∼ 110.0 MYA [16,17]. They subsequently underwent diversification at ∼ 63.4 MYA and further differentiation at 46.7 MYA (Figure 1B, Figure S1). Furthermore, genes related to the terrestrial characteristics of roots, stomata, and phytohormones gradually reduced along the evolutionary sequence of duckweeds (Figure 1B).

Cretaceous–Paleogene (K–Pg) extinction (66.0 MYA) is one of the most important events in the evolution of life on Earth. It provided empty ecological niches and induced adaptive radiation to numerous organisms [18–20]. For example, the extinction of dinosaurs and the emergence of birds are closely related to the evolution of adaptive radiations [21,22]. Interestingly, duckweeds differentiated into *Spirodela* (63.4 MYA) after K–Pg extinction, followed successively by *Landoltia*, *Lemna*, *Wolffiella*, and *Wolffia* (Figure 1A). The evolution of duckweeds is also possibly driven by adaptive radiations after K–Pg extinction.

### Root traits and development

#### Disappearing roots in duckweeds

The emergence of roots during the Devonian Period (416–360 MYA) was essential for plants to colonize the land [23]. Sophisticated root system [24] in land plants functions in soil anchorage, gravity sensing, and acquisition of nutrients and water [4]. Duckweeds are closely related to the Araceae family, whose terrestrial basal lineage, *S. renifolius* [17] (Figure S1), has large root systems [25]. Interestingly, the fossils of the duckweed ancestor *L. scutatum* indicated that it was also a floating aquatic angiosperm with structures like stolon, numerous adventitious roots [26–31], some bore stout primary roots (3 mm wide), and lateral roots [12,32]. These traits represent a transitional state between terrestrial ancestor (Figure S2) and extant duckweeds. The root traits of extant duckweeds are highly reduced compared with the terrestrial basal group of Araceae and the fossil ancestor of duckweeds. Some studies proved weakened root absorption in duckweeds and suggested anchorage as the main function [33]. The number of genes related to root development in duckweeds (Table S2) is not only remarkably reduced compared with *A. thaliana*, *O. sativa*, and *Z. mays*, but also gradually contracted along the evolutionary sequence of the three duckweed species (Figure 1B). *S. polyrhiza*, *L. punctata*, and *L. minor* have 50, 38, and 25 genes that positively regulate root development, and 18, 17, and 23 genes that negatively regulate root development (Figure 1B). Moreover, the expression levels of these positive genes are generally higher in *S. polyrhiza* than in *L. punctata* and *L. minor* (Figure S3; Table S2).

**Figure 2:**
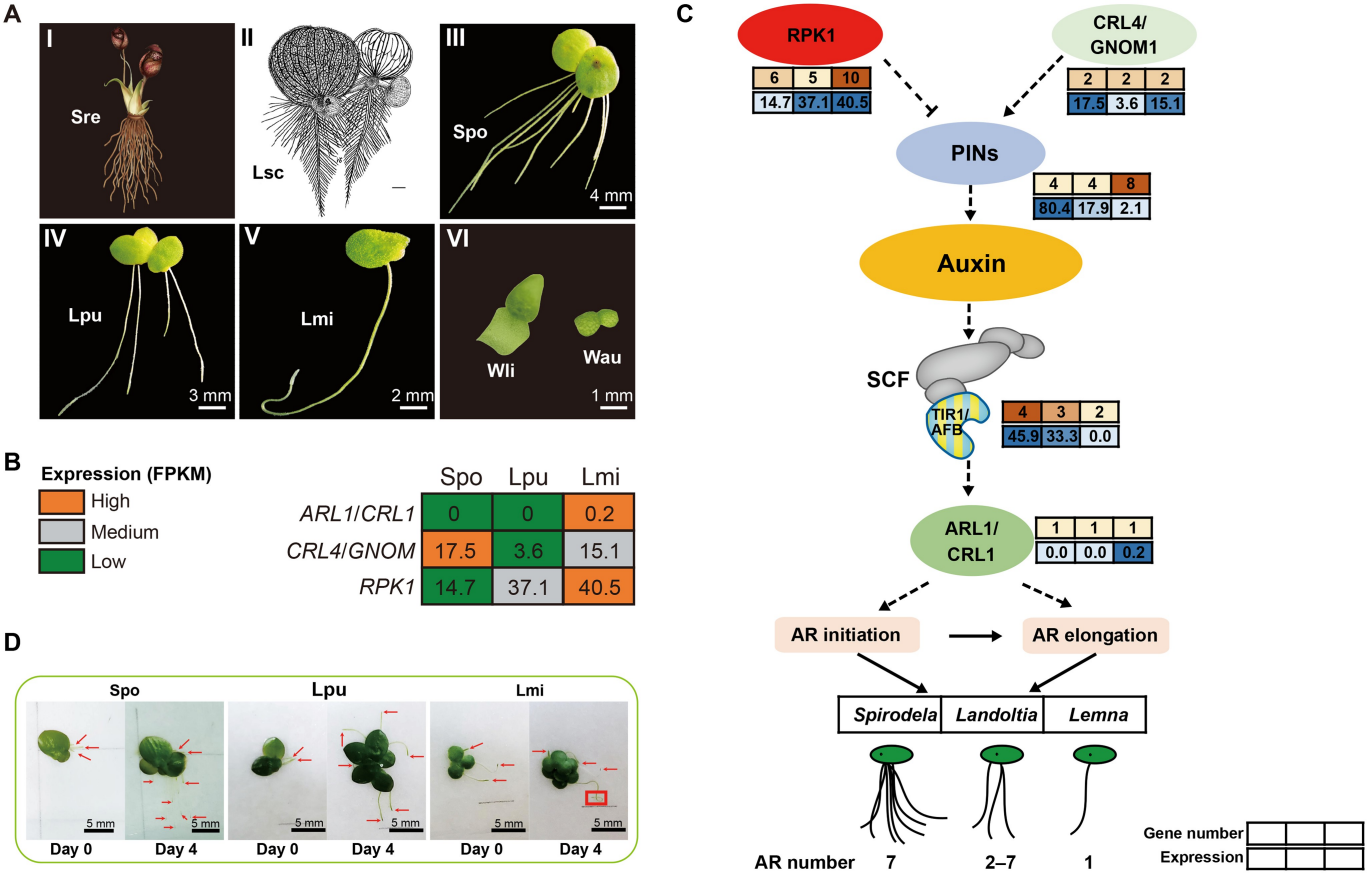
Genetic regulation and physiological characteristics of roots and the cross-talking between auxin and adventitious root development in duckweeds **A**. Root number and morphology of *Symplocarpus renifolius* (I), the fossil of *Limnobiophyllum scutatum* (ancestor of the duckweed family) (II), and extant duckweeds (III–VI). **B**. Expression of key genes involved in adventitious root emergence and development of duckweeds. **C**. Combined effects of auxin signaling-related and rhizoid development-related genes on duckweed adventitious root formation. Heatmaps next to genes represent the corresponding gene number (upper panel) and its expression level (FPKM, lower panel). For the SCF complex genes, only the number and expression of *TIR1/AFB* are shown. Arrows and dotted arrows represent positive regulation, and flat-ended dotted lines indicate negative regulation. **D**. Gravitropic responses of Spo, Lpu, and Lmi roots at different cultivation time points. Red arrows indicate duckweed roots. The direction of gravity is vertically downward. Sre, *Symplocarpus renifolius*; Lsc, *Limnobiophyllum scutatum*; Spo, *Spirodela polyrhiza*; Lpu, *Landoltia punctata*; Lmi, *Lemna minor*; Wli, *Wolffiella lingulata*; Wau, *Wolffia australiana*; FPKM, fragments per kilobase of transcript per million mapped fragments; SCF, Skp1/Cullin/F-box; AR, adventitious root.

Extant duckweeds only have adventitious roots. The numbers of roots in different duckweed genera decrease as follows: 7 roots in *Spirodela*, 2–7 roots in *Landoltia*, only 1 root in *Lemna*, and no root in *Wolffiella* and *Wolffia* (**Figure 2**A). The key genes related to adventitious root emergence and development have undergone a contraction (*ARL1*/*CRL1* and *CRL4*/*GNOM1*) or been lost (*NAL1*) in duckweeds (**Table 1**). The *ARL1*/*CRL1* genes were barely expressed in duckweeds, while the *CRL4*/*GNOM1* genes were more highly expressed in *S. polyrhiza* than in *L. punctata* and *L. minor* (Figure 2B). In addition, the number of *RPK1* genes (encoding a negative regulator) in *S. polyrhiza* and *L. punctata* was similar to that in *A. thaliana*, *O. sativa*, and *Z. mays*, while this number was expanded in *L. minor* (Table 1). Furthermore, *RPK1* in *L. minor* showed the highest expression level amongst the three species (Figure 2B; Table S3). Therefore, the number and expression of key genes which promote adventitious root development gradually decrease along the evolution direction of duckweeds, confirming the trend of diminishing adventitious root in duckweeds.

**Table 1:** Number of key genes involved in adventitious root emergence and development in duckweeds and other plants

Gene	Gene number							
	Kfl	Ath	Osa	Zma	Zos	Spo	Lpu	Lmi
*ARL1*/*CRL1*	0	2	2	2	1	1	1	1
*CRL4*/*GNOM1*	2	3	3	4	2	2	2	2
*NAL1*	0	0	1	0	0	0	0	0
RPK1*	0	5	6	5	1	6	5	10

*Note*: “*” indicates negative regulation. Kfl, *Klebsormidium flaccidum*; Ath, *Arabidopsis thaliana*; Osa, *Oryza sativa*; Zma, *Zea mays*; Zos, *Zostera marina*; Spo, *Spirodela polyrhiza*; Lpu, *Landoltia punctata*; Lmi, *Lemna minor*.

Auxin responses in land plants play an essential role in shaping their morphological traits adapted to terrestrial habitats, particularly in root development and organized three-dimensional growth. The emergence and expansion of the auxin response system align well with the morphological changes controlled by auxin [34]. The highly degenerated and almost two-dimensional structures in duckweeds imply the contraction of their auxin response system. Along the evolution direction of Lemnaceae, the number and expression of the auxin receptor genes *T1R1/AFB* decrease, while those of the negative regulatory gene *RPK1* increase. The number of *PIN* genes in *L. minor* (8) is higher than that in *S. polyrhiza* (4) and *L. punctata* (4), and their expression levels remarkably decrease along the evolution direction [fragments per kilobase of transcript per million fragments mapped (FPKM) = 80.4, 17.9, 2.1, respectively] (Figure 2C; Table S3). These findings suggest that the cross-talks between auxin-related and root development-related genes lead to a gradual decrease in root number and a gradual weakening of root function during the process of migrating back to water (Figure 2C).

#### Loss of lateral roots and root hairs in duckweeds

Lateral roots and root hairs are absent in extant duckweeds, but fossil records of *L. scutatum*, the ancestor of duckweeds, proved their previous existence [12,32]. The genes or transcription factors (TFs) related to the formation of lateral roots and root hairs are lost (*LBD16*, *CPC*, and *WER*) or contracted (*WAK*, *LBD29*, *GL3*, and *GL2*) (Table S2) in these three species. Although previous studies on *S. polyrhiza* and rice have focused on the genes related to phytohormone signal transduction in lateral roots and during root hair elongation [33,35], this study specifically analyzed the loss and contraction of key genes involved in lateral root formation and root hair epidermal cell differentiation in these three duckweed species.

#### Loss of gravitropic responses in *L. minor*

Generally, roots of land plants grow downward in response to gravity to facilitate soil anchorage [36]; however, the roots of *L. minor* grow upward (Figure 2D). Although all three duckweed species possess three regulatory genes (*PLC1*, *WAV3*, and *AGR*) important for gravitropic responses, the number of *PLC1* genes is reduced from 4–7 in model plants to 2 (Table S2). More importantly, expression of these genes was not detected in *L. minor* (Figure S3), which may explain the loss of its gravitropic responses (Figure 2D).

### Stomatal distribution and development

Plant stomata facilitate adaptation to terrestrial environments through their function of optimizing gas exchange and limiting evaporative loss [2,26]. The earliest land plants lacked stomata and were restricted to moist habitats. Around 400 MYA, stomata first appeared in shade-tolerant terrestrial species such as vascular plants, preceding the flourishing of land plants [29]. Therefore, stomata represent an important trait for transition to land.

**Figure 3:**
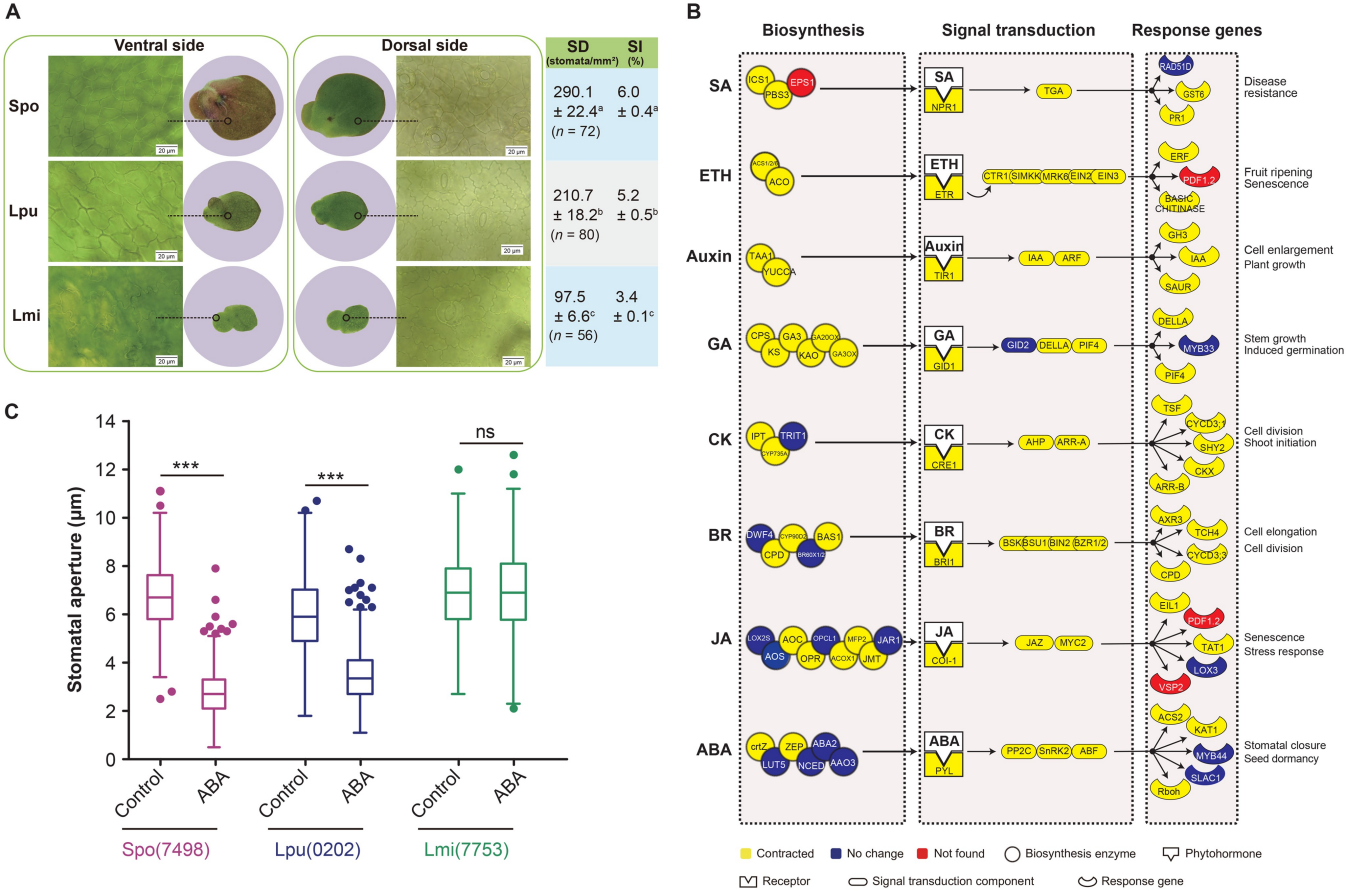
Physiological characteristics of stomata and phytohormone pathways in duckweeds **A**. Stomatal distribution on dorsal and ventral sides of duckweed fronds. Stomata were monitored on the whole duckweed fronds. “*n*” represents the total number of surveyed stomata. Values are represented as mean ± SD. Letters (a, b, and c) indicate significant differences determined by one-way ANOVA followed by multiple comparison with Tukey–Kramer test (*P* < 0.05). Scale bar, 20 μm. **B**. Comparison of phytohormone pathways between duckweeds and typical land plants. Mean numbers of genes in *S. polyrhiza*, *L. punctata*, and *L. minor* were compared to those in *A. thaliana*, *O. sativa*, and *Z. mays*. The number of genes was obtained from the gene family analysis via OrthoMCL. The compared genes were involved in biosynthesis, signal transduction, and response of phytohormones. Detailed information about the gene number and gene ID is provided in Table S5. **C**. Comparison of ABA-induced stomatal closure in *S. polyrhiza* [Spo(7498)], *L. punctata* [Lpu(0202)], and *L. minor* [Lmi(7753)]. Data are represented as mean ± SD. Statistical difference was determined by one way ANOVA (***, *P* < 0.001; ns, not significant). SD, stomatal density; SI, stomatal index; SD, standard deviation; ANOVA, analysis of variance; ABA, abscisic acid; SA, salicylic acid; ETH, ethylene; GA, gibberellic acid; CK, cytokinin; BR, brassinosteroid; JA, jasmonic acid.

Stomatal density (SD) and stomatal index (SI) are good indicators of a plant’s adaptation to its environment [31,37]. Duckweed stomata exist only on the dorsal surface (**Figure 3**A), differing from most land plants which have stomata on both leaf surfaces (ventral and dorsal) [30]. During their transition out of water, duckweeds’ stomata were also under relaxed selection. *S. polyrhiza*, *L. punctata*, and *L. minor* reached SD values of 290.1, 210.7, and 97.5 stomata/mm^2^, and achieved SI values of 6.0%, 5.2%, and 3.4%, respectively (Figure 3A). Both SD and SI exhibited a similar declining trend across these three species. The number of genes involved in stomatal development in these three species exhibited a similar trend to their SD and SI (Figure 1B; Table S4). For example, the number of genes encoding TF SCRM, one of the most important TFs in stomatal lineage progression, decreased to one copy in *L. punctata* and *L. minor*, and two copies in *S. polyrhiza*. Likewise, gene copies of *EPFL9* (a positive regulator of stomatal complex development) and *CDKB1;1* (involved in guard mother cell differentiation) decreased to one in *S. polyrhiza* and *L. punctata* and zero in *L. minor* (Table S4). Compared to *S. polyrhiza* and *L. punctata*, *L. minor* has many more genes suppressing stomatal generation with higher expression levels, such as *SDD1* and *MPK3/MPK6* (Figure S4). These genomic and transcriptomic results are consistent with the morphological changes of stomata within the Lemnaceae family.

### Phytohormone pathways

#### Contraction of phytohormone pathways

Terrestrial environments are more complicated and variable than aquatic ones. Phytohormones in plants play a primary role in adapting to the complex land environments [38]. Although aquatic algae already possessed complete biosynthesis pathways for major phytohormones before migrating to land [39], their signaling pathways remained incomplete. The earliest land plants such as charophytes and liverworts (*K. flaccidum*, *Chara braunii*, and *Marchantia polymorpha*) first expanded these biosynthesis pathways and started to complete the signaling pathways [5,40,41]. Both processes were concluded with the emergence of angiosperms. Duckweeds possess the complete biosynthesis pathways, signal transduction pathways, and response genes of eight major phytohormones: abscisic acid (ABA), salicylic acid (SA), jasmonic acid (JA), ethylene (ETH), auxin, gibberellic acid (GA), cytokinin (CK), and brassinosteroid (BR) (Figure 3B, Figure S5; Table S5). All listed phytohormones were detectable (Figure S5). Baggs et al. [42] have reported that despite the absence of the ENHANCED DISEASE SUSCEPTIBILITY 1 (EDS1) pathway, duckweeds retain the capability to regulate immune defenses against pathogens via phytohormone signaling, particularly SA and JA. However, compared with that in land angiosperms such as *A. thaliana*, *O. sativa*, and *Z. mays*, the number of genes involved phytohormone signaling and responses is remarkably lower in duckweeds (Figure 3B). This contraction presents a sharp contrast to the expansion of the phytohormone pathways in the earliest land plants.

#### Gradual reduction of SCF-mediated phytohormone signaling

Auxin, JA, and GA are three groups of phytohormones which share a common feature in their signaling pathways: the proteolytic targeting of signaling proteins is mediated by the Skp1/Cullin/F-box (SCF) E3 ubiquitin ligase complex. The SCF complexes involved in auxin, JA, and GA signaling first appeared in the land plant lineage [34]. In the alga *K. flaccidum*, which first acquired the fundamental mechanism to adapt to terrestrial environments, only JA signaling involves a complete set of SCF complex genes, while both auxin and GA signaling lack the genes encoding F-box proteins. Meanwhile, *K. flaccidum* harbors only 6, 7, and 6 SCF complex genes for the auxin, JA, and GA signaling pathways, respectively (**Table 2**). During the terrestrialization of plants, the number of SCF complex genes gradually increased, especially in the auxin response system. Although duckweeds have a complete set of SCF complex genes for auxin, JA, and GA signaling, the gene numbers have contracted significantly compared to those in land plants. For example, the numbers of SCF complex genes for auxin signaling are 12, 9, and 8 in *S. polyrhiza*, *L. punctata*, and *L. minor*, respectively. These numbers are remarkably lower than those in *A. thaliana* (35), *O. Sativa* (31), and *Z. mays* (29) and exhibit a gradual reduction along the evolution direction of the three duckweed species (Table 2, Table S6). Similarly, the numbers of SCF complex genes for JA and GA signaling in these three duckweeds are also remarkably reduced compared to the land plants (*A. thaliana*, *O. sativa*, and *Z. mays*) and gradually decline along the evolutionary direction of these three species (Table 2, Table S6).

**Table 2:** Number of key genes involved in SCF-mediated phytohormone signaling of auxin, JA, and GA

Phytohormone	SCF complex gene		Gene number							
			Kfl	Ath	Osa	Zma	Zos	Spo	Lpu	Lmi
Auxin	*SKP1*		4	22	21	16	3	4	3	3
	*RBX1*		1	2	2	3	2	2	2	2
	*CUL1*		1	5	3	2	2	2	1	1
	*TIR1/AFB* (F-box protein)		0	6	5	8	3	4	3	2
	Total		6	35	31	29	10	12	9	8
JA	*SKP1*		4	22	21	16	3	4	3	3
	*RBX1*		1	2	2	3	2	2	2	2
	*CUL1*		1	5	3	2	2	2	1	1
	*COI-1* (F-box protein)		1	1	3	6	3	2	2	1
	Total		7	30	29	27	10	10	8	7
GA	*SKP1*		4	22	21	16	3	4	3	3
	*RBX1*		1	2	2	3	2	2	2	2
	*CUL1*		1	5	3	2	2	2	1	1
	*GID2/SLY1* (F-box protein)		0	1	0	2	1	1	1	1
	Total		6	30	26	23	8	9	7	7

*Note*: JA, jasmonic acid; GA, gibberellic acid; SCF, Skp1/Cullin/F-box.

#### Gradual weakening of ABA-induced stomatal closure

The crucial function of ABA in land plants is to mediate drought stress responses and is mainly achieved by inducing stomatal closure [43]. This ABA-induced stomatal closure emerged in early land plants such as mosses and lycophytes and were further developed in ferns [44]. The stomata of typical angiosperms can close completely under ABA treatment.Although ABA-induced stomatal closure exists in duckweeds, it weakens gradually along the evolution direction of the three species. Surprisingly, unlike other typical angiosperms, the stomata of *S. polyrhiza* and *L. punctata* cannot close completely in response to ABA (Figure 3C). This trait is also observed in an early land moss (*Physcomitrella patens*) [45]. More importantly, the degree of stomatal closure in response to ABA treatment decreases in the order of *S. polyrhiza* (stomatal aperture from 6.7 ± 1.4 μm to 2.8 ± 1.0 μm), *L. punctata* (stomatal aperture from 6.0 ± 1.6 μm to 3.5 ± 1.2 μm), and *L. minor* (stomatal aperture from 6.9 ± 1.4 μm to 6.9 ± 1.8 μm). In fact, the species in the genus *Lemna* were completely unaffected by ABA treatment (Figure 3C, Figure S6).

The alga *K. flaccidum* has no stomata and no complete gene set (only 5 genes) for ABA-induced stomatal closure signaling pathway. Evolutionarily, the gene number in this pathway peaked with the emergence of angiosperms, reaching 68, 63, and 81 genes in *A. thaliana*, *O. sativa*, and *Z. mays*, respectively (**Table 3**). Compared with land plants, the gene numbers of this pathway in all three duckweed species have contracted, following their evolutionary sequence (33, 29, and 24, respectively) (Table 3, Table S7). This indicates that in duckweeds, the decreasing trend of gene numbers in the ABA-induced stomatal closure signaling pathway is consistent with the degree of stomatal closure observed in these three species.

**Table 3:** Number of key genes involved in ABA-induced stomatal closure signaling in eight typical species

Gene	Gene number							
	Kfl	Ath	Osa	Zma	Zos	Spo	Lpu	Lmi
*PYL*	0	14	13	12	6	4	4	2
*PP2C*	1	9	8	14	3	5	4	4
*M3K*	0	3	3	3	1	1	1	1
*RAF B2/3/4*	2	19	15	18	9	11	8	7
*SnRK2* II	0	2	3	3	0	2	2	0
*SnRK2* III	1	3	3	4	1	1	1	1
*ABF*	1	11	11	16	5	5	6	5
*KAT1*	0	6	6	10	4	3	2	3
*SLAC1*	0	1	1	1	1	1	1	1
Total	5	68	63	81	30	33	29	24

*Note*: ABA, abscisic acid.

In the three duckweed species, the number of genes involved in major phytohormone pathways significantly contracted, contrary to the expansion trend in land plants. In particular, along the evolutionary sequence of duckweeds, not only the stomatal development but also the stomatal response to ABA treatment gradually weakened from *S. polyrhiza* to *L. punctata* and to *L. minor*.

### Lignin, cellulose, and hemicellulose

#### Low lignin content

**Figure 4:**
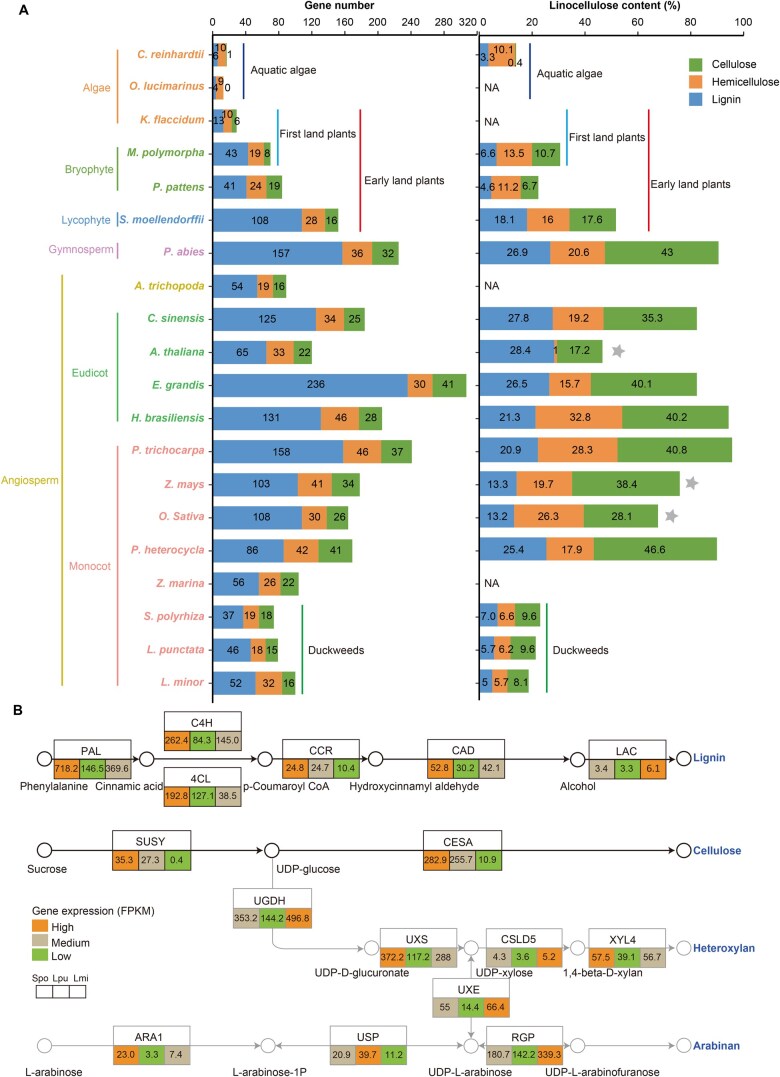
Content, gene number, and expression for cellulose, hemicellulose, and lignin in duckweeds and other plants **A**. Content of cellulose, hemicellulose, and lignin as well as the number of genes involved in their biosynthesis pathways in duckweeds and other plants (Tables S8–S11). Gray stars indicate data available from plant stems. “NA” indicates no available measurement data. **B**. Expression levels (FPKM) of genes encoding key enzymes involved in the biosynthesis pathways of cellulose, heteroxylan, arabinan, and lignin in duckweeds. Detailed gene information and expression data are provided in Tables S9–S11.

Cellulose, hemicellulose, and lignin are important biopolymers assisting land plants to resist gravity, grow vertically, and transport water [3,46]. Lignin appeared in land plants around 400 MYA and is considered an essential evolutionary biomarker for terrestrial adaptation [3]. Lignin content was initially extremely low (3.3%, w/w) in aquatic algae, then increased in early land plants such as liverworts (*M. polymorpha*) and mosses (*P. patens*) (4.6%–6.6%, w/w), and eventually reached over 20% in terrestrial angiosperms. However, lignin content in duckweeds is below 7%, much lower than that in other typical angiosperms (**Figure 4**A; Table S8).

The number of genes involved in lignin biosynthesis expanded from lower aquatic algae (*e.g.*, 6 in *Chlamydomonas reinhardtii* and 4 in *Ostreococcus lucimarinus*) to early land plants (*e.g.*, 43 in *M. polymorpha* and 41 in *P. patens*) (Figure 4A; Table S9). However, these plants lack *F5H*, the key gene for biosynthesis of S lignin [47], and have only few *CCR*, *CAD*, and *LAC* copies (Table S9). This leads to an incomplete lignin biosynthesis pathway and therefore low lignin content (Figure 4A). In contrast, duckweeds, as embryophytes, have a complete lignin biosynthesis pathway that includes *F5H*, though their *F5H* copy number remains less than that in typical land plants. Nevertheless, the total number of genes involved in the lignin biosynthesis pathway in duckweeds has contracted to a level comparable to that of early land plants (Figure 4A), despite differences in their genetic structures.

Laccase (LAC) is another important enzyme involved in the final step of lignin biosynthesis for polymerization [48,49]. The copy number of *LAC* genes decreased from 17–74 in typical land plants to 5–8 in duckweeds (Table S9). In the LAC gene family, *LAC4*/*11*/*17* are essential for lignin formation in *Arabidopsis*, and mutations of these genes lead to different degrees of low lignin content [49]. All three duckweed species lack *LAC4*. Both *LAC11* and *LAC17* are present in *S. polyrhiza*, whereas only *LAC11* is found in *L. punctata* and *L. minor* (Figure S7). Nonetheless, the expression levels of *LAC11* and *LAC17* in duckweeds are extremely low (Figure 4B; Table S9). The reduction in both copy number and expression of lignin-related genes is consistent with the decreasing trend of lignin content in the three species in the order of *S. polyrhiza* (7.0%), *L. punctata* (5.7%), and *L. minor* (5.0%) (Figure 4A).

Therefore, genomic and transcriptomic evidence strongly supports the low lignin content in duckweeds and its decreasing trend along the evolutionary sequence.

#### Low cellulose and hemicellulose content

Holocellulose content in duckweeds (13.8%–16.2%) is much lower than that in typical land plants (18.2%–73.0%) (Table S8). The number of genes involved in holocellulose biosynthesis in duckweeds (33–48 genes) is reduced compared to that in typical land plants (35–83 genes) (Figure 4A; Tables S10 and S11). Furthermore, the expression levels of key cellulose biosynthesis genes, including the precursor gene *SUSY* and the cellulose synthase gene *CESA*, align with the trend of cellulose content in the three duckweed species (Figure 4B).

In summary, despite possessing a complete lignocellulose biosynthesis pathway, duckweeds have undergone a contraction in the copy number of associated genes to levels comparable to those of early land plants (*e.g.*, liverworts and mosses). This might be the result of the degeneration of lignocellulose and its function in duckweeds to adapt to the new aquatic environment.

### Drought stress response

#### Contraction of *LEA* genes

Drought is one of the major abiotic stresses that affects the growth of land plants. As a result, various regulatory systems against drought stress have evolved [50,51]. Late embryogenesis abundant (LEA) proteins are hydrophilic proteins involved in abiotic stress tolerance, especially in drought response [51]. The number of *LEA* genes has contracted in duckweeds (13–19 genes) compared to typical land plants (18–52 genes), especially in genes encoding dehydrins (1–2 genes in duckweeds) which belong to LEA group II and are mainly involved in drought resistance (Table S12). Moreover, duckweeds lack the ortholog of the *Polypedilum vanderplanki LEA18* (*PvLEA18*) gene. This gene is also absent in the marine angiosperm *Z. marina* which belongs to the same order Alismatales as duckweeds [9] (Figure S8). These findings imply that aquatic plants no longer need a strong drought resistance ability, resulting in a significant contraction of their drought resistance genes.

#### Contraction of TF gene families bHLH, C2H2, and WRKY

TF gene families expanded drastically during plant terrestrialization. The aquatic alga *C. reinhardtii* possess 208 TF genes, the first-landing liverwort *M. polymorpha* has 398, and the land dicot *A. thaliana* has 1780 [40]. In comparison, the three duckweed species have 1076–1148 TF genes (Table S13), positioned between the first-landing liverwort and *A. thaliana*. The TF gene families of bHLH, C2H2, and WRKY play important roles in response to abiotic stresses. These three families have contracted significantly in duckweeds, presumably because of the relatively stable aquatic environment (Figure S9A; Table S14). Especially, the TF genes in these three families that are known to respond to drought stress have contracted (*e.g.*, *AtWRKY28/57*, *OsWRKY07*, and *OsWRKY30*) or been lost (*e.g*., *AtZAT12* and *OsZFP182*) in duckweeds (Figure S9B).

The contraction of *LEA* genes and TF gene families bHLH, C2H2, and WRKY suggests that during the transitional process from terrestrial to aquatic habitats, the regulatory networks related to drought response in duckweeds have declined.

### Expanded gene families in duckweeds

#### Expansion of gene families in the flavonoid biosynthesis pathway

Flavonoids play an important role in plant defense against bacteria, fungi, and viruses [52]. Plant flavonoids originate from charophytic algae, hypothetically closely related to land plants, to adapt to terrestrial environments with more adversity [5,53].

*K. flaccidum* lacks a complete flavonoid biosynthesis pathway, while the gene numbers of these pathways in land model plants *A. thaliana*, *O. sativa*, and *Z. mays* have increased significantly and formed complete pathways [40,54,55]. Compared with these three model plants, the most prominently expanded pathway in duckweeds is flavonoid biosynthesis, markedly expanded in all three species. The flavone and flavonol biosynthesis pathway has expanded in *S. polyrhiza* and *L. punctata*, and the anthocyanin biosynthesis pathway has expanded in *S. polyrhiza* and *L. minor* (Figure S10). The gene numbers of expanded gene families involved in flavonoid-related pathways (flavonoid, anthocyanin, and flavone and flavonol biosynthesis) in *S. polyrhiza* (43), *L. punctata* (63), and *L. minor* (33) are much higher than those in land model plants (Table S15). Moreover, both flavonoid and anthocyanin content in duckweeds are extremely high, reaching 3.7%–5.5% and 0.21%–0.93% of their dry weight, respectively (Figure S11A and B), significantly higher than those in other plants [56–59]. In addition, flavonoids in duckweeds exist in a large diversity (more than 30 compounds in *L. punctata*) (Figure S11C).

Flavonoids are also known to assist in maintaining human health, especially in the prevention and treatment of inflammation, heart disease, and cancer [60]. Duckweeds have high flavonoid content and abundant diversity, as well as the expansion of related biosynthetic gene families. Therefore, duckweeds have great potential as a new source of flavonoids [61]. In fact, some human trials have already been carried out with valuable positive results [62].

Notably, the flavonoid and lignin biosynthesis pathways share the same precursor phenylalanine pathway. Compared with land plants, the number of genes in the phenylalanine pathway in duckweeds is slightly reduced (Table S16). When comparing its downstream pathways, the lignin biosynthesis pathway in duckweeds is extremely reduced (Table S9), while the flavonoid biosynthesis pathway has significantly expanded (Table S15). These sharp changes are possibly adaptations to their new aquatic environment. Meanwhile, flavonoids act as ultraviolet (UV) “sunscreens” in land plants and are boosted by UV-B [40]. Floating in water with direct exposure to the sun, duckweeds don’t need much lignin for structural support but require more flavonoids for stronger antibacterial ability and UV resistance. This also explains why duckweeds can grow vigorously in eutrophic wastewater and can be completely exposed to high levels of UV radiation.

#### Expansion of regulatory genes

As key editing factors, pentatricopeptide repeat (PPR) proteins bind to organellar transcripts and influence their expression by altering RNA sequence, stability, processing, or translation [5,63]. The two duckweed species, *S. polyrhiza* and *L. punctata,* have more *PPR* genes (502–579) than the three model land plants, *A. thaliana*, *O. sativa*, and *Z. mays* (407–458) (Table S17). This expansion suggests stronger transcriptional regulatory ability in duckweeds and explains its flexible environmental adaptability with such small genome size.

Transposable elements (TEs) are associated with the enhancement of regulatory ability, adaptability, and diversification [53,58]. Among the three duckweeds, the proportion of TEs gradually increases along the evolutionary direction (14.7% in *S. polyrhiza*, 52.5% in *L. punctata*, and 61.5% in *L. minor*) (Table S18). This gradient in TE levels may explain why *Lemna* is more widely distributed than *Spirodela* and *Landoltia* on a global scale.

## Conclusion

Due to fragmented fossil records and unreliable phylogenetic trees based on merely a few genes, only limited information on the differentiation of existing species and their ancestors can be excavated in evolutionary studies. It is almost impossible to reconstruct the evolutionary process in detail. Herein, we studied three extant duckweed species and their ancestral fossils. Our results demonstrated a pattern of progressive evolution, characterized by the gradual reduction of several important traits associated with terrestrial adaptation. These include stomatal formation and movement, lignin content, root development, phytohormone pathways, and the gradual contraction of their corresponding genes. Collectively, these findings reconstruct the process by which a land plant returned to water, an opposite direction of plant terrestrialization different from textbook definition (Figure 1A). This finding could deepen and widen our understanding of plant adaptation while shaping our perspective on evolutionary theories.

## Materials and methods

### Sample preparation and genetic analysis

*L*. *punctata* strain 0202 was collected and stored at the Chengdu Institute of Biology, Chinese Academy of Sciences, Chengdu, China. *S*. *polyrhiza* strain 7498 and *L*. *minor* strain 7753 were gifts from the Department of Plant Physiology at the Matthias Schleiden Institute, University of Jena, Germany. They were originally obtained from Sichuan (China), North Carolina (USA), and Hara (Ethiopia), respectively.

One plant of each stored strain was inoculated into 100 ml of sterilized Hoagland medium supplemented with 15 g/l sucrose (pH 5.0), and then cultivated at 25°C ± 2°C under a 16-h/8-h light/dark cycle with a light intensity of 110 μmol·m^−2^·s^−1^ in a climatic chamber. Once the duckweed plants covered the medium surface completely, they were transferred into 1/5 Hoagland medium (pH 5.5) and further cultivated for 5 days at 25°C ± 2°C under the same temperature and light conditions. The cultivated plants were used for the subsequent genome and transcriptome sequencing, as well as for physiological and biochemical analyses.

### Genome sequencing, assembly, and annotation

#### Chromosome observation

The chromosome number of *L*. *punctata* was determined by 4′,6-diamidino-2-phenylindole (DAPI) staining as described previously [64]. This advanced cytogenetic method has been successfully established, particularly for small-sized chromosomes, such as those in duckweed species [65].

#### Genome sequencing

Genomic DNA of *L*. *punctata* strain 0202 was extracted using a modified cetyltrimethylammonium bromide (CTAB) method [66]. DNA concentration and purity were determined using NanoDrop 2000c (Thermo Fisher Scientific, Waltham, MA). Paired-end libraries with insert sizes of 200 bp, 500 bp, and 800 bp and mate-pair libraries with insert sizes of 2 kb, 5 kb, 10 kb, and 20 kb were constructed and sequenced on the Illumina HiSeq 2000 platform at the Beijing Genomics Institute (Shenzhen), China.

#### Genome assembly

The genome size of *L*. *punctata* was estimated based on K-mer frequency distribution analysis. Briefly, the size was calculated using the following formula: genome size = K_num_/K_depth_, where K_num_ is the number of K-mers and K_depth_ is the expected depth of K-mers. The clean reads from the 200 bp, 500 bp, and 800 bp insert-size libraries were subjected to 17-mer analysis. Prior to assembly, potential sequencing errors in the short-insert paired-end libraries were removed or corrected using the K-mer frequency methodology. The genome was assembled using SOAPdenovo (v2.0) [67] with a K-mer of 81 and scaffolded using SSPACE (v2.0.2) [68]. Gaps in the scaffolds were closed using GapCloser (v1.12). Scaffolds shorter than 150 bp were removed. To eliminate bacterial contamination, scaffolds were screened against the National Center for Biotechnology Information (NCBI) database. Assembly quality was assessed using Benchmarking Universal Single-Copy Orthologs (BUSCO) [69] and Expressed Sequence Tags (EST) methods. The scaffolds were aligned to the genome of *S*. *polyrhiza* strain 9509 (Sp9509) [70] using Local Alignment Search Tool (LAST; http://last.cbrc.jp/) with E-value ≤ 1.0 × 10^−5^, retaining only the best-scoring match in cases of multiple matches. Finally, the genomic sequences of *L*. *punctata* were anchored to 20 chromosomes by identifying syntenic blocks with *S*. *polyrhiza* strain 9509 using Allele-aware Scaffold Integration and Ordering (ALLMAPS) [71].

#### Genome annotation

##### Repetitive sequence annotation

We detected the repetitive sequences in the genome by combining homology-based and *de novo* approaches. Homology-based approach used Tandem Repeats Finder (TRF) [72], RepeatMasker (v4.0.3), and RepeatProteinMask (v3; http://www.repeatmasker.org/) against the RepBase database [73]. The *de novo* repeats were identified using RepeatModeler (v1.0.7; https://github.com/Dfam-consortium/RepeatModeler) and annotated using RepeatMasker (v4.0.3) [74]. The repetitive sequences predicted by the two approaches were combined and the redundancies were removed.

##### Gene prediction

All repetitive sequences were masked in the genome prior to gene prediction. Genes were subsequently predicted by combining homology-based, *de novo*, and transcript-based approaches. For homology-based prediction, protein sequences from *A*. *thaliana*, *Brachypodium distachyon*, *Z*. *mays*, *O*. *sativa*, *Sorghum bicolor*, *L*. *minor*, and *S*. *polyrhiza* were aligned to the *L*. *punctata* strain 0202 genome using Translated Basic Local Alignment Search Tool for Nucleotide (TBLASTN) [75] with E-value ≤ 1 × 10^−5^, to identify putative coding regions. For *de novo* prediction, AUGUSTUS (v2.5.5; http://augustus.gobics.de/) and GENSCAN [76] were applied using gene model parameters trained on *A*. *thaliana*. For transcript-based approach, TopHat (v2.1.0) [77] and Cufflinks (v2.2.0) [78] were used to assemble transcripts, and the fifth-order Markov model was used to predict open reading frames (ORFs). All available ESTs from *L*. *punctata* were used to predict genes. Finally, a combined gene set was generated using GLEAN (https://sourceforge.net/projects/glean-gene/) with default parameters, and the redundancies were removed.

##### Functional annotation of genes

Gene Ontology (GO) terms [79] and Kyoto Encyclopedia of Genes and Genomes (KEGG) pathways [80] were annotated using an InterProScan analysis [81]. Gene functional descriptions were generated through sequence homology search against InterPro (http://www.ebi.ac.uk/interpro/) and UniProtKB/Swiss-Prot [82] databases using Basic Local Alignment Search Tool for Protein (BLASTP) [83].

### Gene family analyses

Protein sets were collected from eight species: *K*. *flaccidum* [41], *Z*. *marina* [9], *A*. *thaliana* [84], *O*. *sativa japonica* [85], *Z*. *mays* [86], *S*. *polyrhiza* [87], *L*. *punctata*, and *L*. *minor* [88]. These sequences were subjected to an “all-versus-all” BLASTP comparison (E-value ≤ 1 × 10^−3^), followed by gene family delineation using OrthoMCL [89] (with parameter “mcl –I 1.5”). Next, gene families involved in root and stomatal development, phytohormone pathways, TFs, and PPR proteins were summarized. In addition, to analyze gene families involved in the lignocellulose biosynthesis pathway, protein sets from 23 species were collected and analyzed using OrthoFinder (v2.2.1) [90] (Tables S9–S11). Moreover, prediction and classification of TF genes of the three duckweeds were conducted using iTAK [91].

### Phylogenetic tree construction and divergence time estimation

For phylogenetic analyses based on the nuclear genome, the amino acid sequences of 1545 single-copy genes shared by the 8 species (*S*. *polyrhiza*, *L*. *punctata*, *L*. *minor*, *O*. *sativa*, *Z*. *mays*, *A*. *thaliana*, *Z*. *marina*, and *K*. *flaccidum*) were aligned using Multiple Sequence Comparison by Log-Expectation (MUSCLE) with default settings. The aligned protein sequences were concatenated into a supergene. Phylogenetic analyses were performed using the maximum-likelihood (ML) method implemented in Phylogeny estimation under Maximum Likelihood (PhyML; v3.0) [92]. Nucleotide substitution model selection was estimated with Smart Model Selection [93] in PhyML (v3.0). The JTT **+** G **+** I **+** F model was selected as the best-fitting model for ML analyses. One thousand bootstrap replicates were performed to calculate the bootstrap values.

For phylogenetic analyses based on the chloroplast genome, sequences from 23 species (*C*. *reinhardtii*, *K*. *flaccidum*, *Marchantia paleacea*, *P*. *patens*, *Selaginella moellendorffii*, *Picea abies*, *Amborella trichopoda*, *Glycine max*, *Medicago truncatula*, *Carica papaya*, *A*. *thaliana*, *Vitis vinifera*, *Populus trichocarpa*, *Z*. *mays*, *O*. *sativa japonica*, *T*. *thibetica*, *S*. *renifolius*, *Colocasia esculenta*, *S*. *polyrhiza*, *L*. *punctata*, *L*. *minor*, *Wolffiella lingulata*, and *Wolffia australiana*) were aligned and concatenated by the HomBlocks pipeline [94] with default settings (--align -method = Gblocks). A ML tree was constructed using IQ-TREE with the GTR + F + R4 model [95]. One thousand bootstrap replicates were performed to calculate the bootstrap values of the topology.

Divergence times on the phylogeny were inferred using the r8s package (v1.70) [96] with the fixage command, which implements a penalized likelihood method. Node divergence times were calibrated based on the TimeTree database (http://www.timetree.org/). Additionally, the KaKs_Calculator toolbox (v2.0) [97] was used to calculate the non-synonymous substitution rate (Ka), synonymous substitution rate (Ks), and Ka/Ks of orthologous genes among the eight species.

### Transcriptome sequencing

Cultivated duckweed fronds were collected and immediately snap-frozen in liquid nitrogen, then stored at −80°C until RNA extraction. Total RNA was extracted using OMEGA E.Z.N.A. Plant DNA/RNA Kit (Catalog No. R6827-00S, OMEGA, Norcross, GA) following the manufacturer’s instructions. RNA concentration, RNA quality, and RNA integrity number (RIN) were measured by Agilent 2100 Bioanalyzer System (Agilent Technologies, Santa Clara, CA). Complementary DNA (cDNA) libraries were constructed through a series of steps including messenger RNA (mRNA) and non-coding (ncRNA) isolation, fragmentation, cDNA synthesis, end repair, A-tailing, adapter ligation, degradation of the second strand, and polymerase chain reaction (PCR) amplification. Qualified libraries were subjected to pair-end sequencing (2 × 150 bp) on the Illumina HiSeq 2500 platform at Gene Denovo (Guangzhou, China). The characteristics of the RNA sequencing (RNA-seq) data are listed in Tables S33 and S34. All raw reads were evaluated using FastQC (v0.11.3). Low-quality reads (those with adapters, > 10% ambiguous “N” bases, or low quality scores) were filtered out. High-quality clean reads were then aligned to a ribosomal RNA (rRNA) database via Bowtie (v2) [98] to remove rRNA-derived reads. The remaining reads were mapped to the reference genome using TopHat (v2.1.0) [77]. Gene expression levels (FPKM) were calculated using Cufflinks [78].

### Physiological and biochemical analyses

#### Morphological observation of duckweeds

Duckweeds cultivated for 3 days were sampled for morphological observation. The morphology of roots and stomata was examined using a Motic BA210 microscope (Motic Microscopes, Richland, WA) equipped with a Motic imaging accessory (Motic Images Advanced v3.2). Stomatal aperture was measured from the acquired images. SD and SI [37] were calculated as follows:


(1) $$\mathrm{SD}\;(\mathrm{stomata}/{\mathrm{mm}}^{2})=\;\frac{\mathrm{Number}\;of\;\mathrm{stomata}\;in\;\mathrm{one}\;\mathrm{microscopic}\;\mathrm{field}}{\mathrm{Area}\;of\;\mathrm{one}\;\mathrm{microscopic}\;\mathrm{field}}$$

(2) $$\mathrm{SI}\;(\%)=\frac{\mathrm{Stomatal}\;\mathrm{density}}{\mathrm{Stomatal}\;\mathrm{density}+\mathrm{epidermal}\;\mathrm{cell}\;\mathrm{density}}\;\times 100$$

#### Stomatal response of duckweeds to ABA treatment

The stomatal response of duckweeds to ABA treatment was assessed as previously described [99]. Briefly, *S*. *polyrhiza*, *L*. *punctata*, and *L*. *minor* were cultivated in 1/5 Hoagland medium for 3 days. After being rinsed with deionized water, the duckweeds were transferred into fresh 1/5 Hoagland medium and placed under white light for 3 h to induce stomatal opening. Then, ABA was added into the medium to a final concentration of 100 μM, and the duckweeds were treated for 60 min, with a group under identical conditions but without ABA treatment as the control. Stomatal aperture was measured before and after ABA treatment. More than 400 stomata were measured in each group, and at least 10 stomata were measured on each frond.

#### Analytical methods

Lignin and structural carbohydrates (including glucan, xylan, galactan, arabinan, and mannan) were determined according to the method recommended by National Renewable Energy Laboratory, USA [100]. Starch was determined according to the procedure based on hydrolysis using HCl, as described in our previous study [101]. The difference between glucan and starch was considered cellulose. The sum of xylan, galactan, arabinan, and mannan was considered hemicellulose.

Phytohormones were extracted from duckweed tissues with 80% methanol (methanol/water, 8:2, v/v) and analyzed using ultra-performance liquid chromatography-electrospray ionization-tandem mass spectrometry (UPLC-ESI-MS/MS). Briefly, freshly harvested duckweed was snap-frozen in liquid nitrogen and stored at −80°C until analysis. Frozen duckweed of 120 mg was ground and extracted overnight with 1.2 ml of 80% methanol at 4°C. After centrifugation at 12,000 *g* at 4°C for 15 min, the supernatant was collected and evaporated to dryness under nitrogen gas stream. The dried residues were redissolved in 100 μl of 30% methanol (methanol/water, 3:7, v/v) and centrifuged again. The supernatant was collected and analyzed using UPLC-ESI-MS/MS (UPLC, Shim-pack ultra-fast liquid chromatography CBM30A system, Shimadzu, Kyoto, Japan; MS/MS, Triple Quad 6500 System, Applied Biosystems, Thermo Fisher Scientific).

#### Gene family expansion analysis in duckweeds

The analysis of gene family expansion in the three duckweeds compared with model plants was based on the results of OrthoMCL analysis. Expanded families were defined as those in which the total number of genes in duckweeds was higher than the total number of genes in the three model plants. GO terms and KEGG pathways were examined for statistically significant enrichment using OmicShare Tools (http://www.omicshare.com/tools) by conducting a hypergeometric test.

#### Identification of *SnRK2 (III)* in *L. minor*

To confirm the precise coding sequence of the SnRK2 (III) protein in *L*. *minor* in our study, the *SnRK2 (III)* coding sequence was cloned for sequencing in *L*. *minor*. Primers were designed based on the *L*. *minor* reference genome published by Van Hoeck and colleagues [88], which annotates this gene under the accession number Lminor_002649. The forward primer is 5′-ATACTGCTCACTCGCCGGGTTC-3′ and the reverse primer is 5′-ACACGCAGCACGGAGGATTCAA-3′. Total RNA of *L*. *minor* was extracted using Eastep Super Total RNA Extraction Kit (Catalog No. LS1040, Promega, Madison, WI) and then reverse-transcribed into cDNA using GoScript Reverse Transcription System (Catalog No. A5000, Promega). The PCR product of the target *SnRK2 (III)* gene was cloned into the pClone007 Versatile Simple Vector (Catalog No.TSV-007VS, Tsingke, Beijing, China) and used for sequencing. As shown in Figure S12, the coding sequence of *SnRK2 (III)* in *L*. *minor* is similar in sequence and length to its orthologs in *S*. *polyrhiza* and *L*. *punctata*. However, the coding sequence of Lminor_002649 annotated in the reference genome has an additional segment in the intermediate region as well as a deletion near the 3′-end region.

#### Gravitropism experiment

The duckweeds *S*. *polyrhiza*, *L*. *punctata*, and *L*. *minor* were separately inoculated closely on filter paper pre-saturated with 1/5 Hoagland medium. Then, the filter paper was vertically placed against the inner wall of a 500-ml beaker containing 450 ml of 1/5 Hoagland medium, keeping the duckweeds 2–3 cm above the liquid surface. Duckweeds were cultivated for 4 days at 25°C under a light intensity of 130 μmol·m^−2^·s^−1^. The morphology of duckweed roots was observed daily.

#### Quantification of total flavonoids in duckweeds

Plants from five duckweed species: *S*. *polyrhiza* (7 ecotypes), *Spirodela intermedia* (3 ecotypes), *L*. *punctata* (2 ecotypes), *L. minor* (3 ecotypes), and *W. australiana* (1 ecotypes) (Figure S11), were cultivated in 50 ml of distilled water in 100-ml flasks for 7 days. The growth conditions were as follows: a light intensity of 130 μmol·m^−2^·s^−1^, a photoperiod of 16-h day at 25°C and 8-h night at 15°C, and 80% relative humidity. After cultivation, the duckweeds were harvested for total flavonoid analysis. The total flavonoid content was quantified by high-performance liquid chromatography with ultraviolet detector (HPLC-UV; SpectraSYSTEMTM AS3000 and UV6000 Detector, Thermo Fisher Scientific) following a previously established method [56]. Three independent biological replicates were analyzed for each sample.

#### Quantification of anthocyanins in duckweeds

Plants from five duckweed species: *S*. *polyrhiza* (7 ecotypes), *S*. *intermedia* (3 ecotypes), *L*. *punctata* (2 ecotypes), *L. minor* (3 ecotypes), and *W. australiana* (1 ecotypes) (Figure S11), were cultivated in 250 ml of 1/5 Hoagland medium under a light intensity of 110 μmol·m^−2^·s^−1^. For anthocyanin quantification, 50 mg of dry duckweed powder was dissolved in 5 ml of 70% methanol (containing 2% formic acid), ultrasonicated for 10 min, and then kept in the dark for 5 h. The resulting crude extract was filtered by a 0.22-μm filter and analyzed by HPLC-UV (SpectraSYSTEMTM AS3000 and UV6000 Detector, Thermo Fisher Scientific). The elution time was 15 min, using a mobile phase of 10% acetic acid (A) and 100% methanol (B) at a flow rate of 0.6 ml/min. Detection was performed at 530 nm, with a scanning range of 200–798 nm. Cyanidin-3-glucoside was used as an external standard for anthocyanin quantification.

#### Quantification of flavonoids in *L. punctata*

Plant powder (0.25 g) was ultrasonically extracted with 5.0 ml of 70% methanol for three times (30 min for each time). The solvent was concentrated to dryness in a rotary evaporator at 60°C under reduced pressure. The residues were dissolved in 1.0 ml of 70% methanol in a volumetric flask and then filtered through a 0.2-μm polytetrafluoroethylene (PTFE) membrane by a syringe filter before use. The flavonoid profile of the crude extract was analyzed by HPLC on an LC CO-100 instrument equipped with a UV6000 DAD detector (Thermo Fisher Scientific), an AXW-8 temperature controller (Thermo Fisher Scientific), and an AS3000 autosampler (Thermo Fisher Scientific), as well as a Kromasil column (Catalog No. 100-5-C18, Nouryon, Amsterdam, Netherlands) and a UniSil 10-100 C18 column (250 mm × 4.6 mm; United Chemical Technologies, Bristol, PA), at a detection wavelength of 326 nm. The UPLC-ESI-Q-TOF-MS2 analysis was performed on a Waters Vion IMS QTof system (Waters, Milford, MA) equipped with a photodiode-array detector PDAeλ (Waters) [102]. Same condition was used for mass spectrometers equipped with a Kromasil 100-5-C18 column as above. High-purity nitrogen (N_2_) was used as the nebulizing gas, and ultra-high-purity helium (He) was used as the collision gas. The ion source was operated in both negative and positive modes. The mass scan arrange was set as *m/z* 50–1000 for TOF MS2 scan. The key parameters were set as follows: ion spray voltage, 3000 V in positive and 2500 V in negative; source temperature, 120°C; desolvation temperature, 450°C; cone gas flow, 50 l/h; desolvation gas flow, 800 l/h; and scan time, 0.200 s. The mobile phase and condition were the same as those described above.

### Statistical analysis

The Wilcoxon rank-sum test was applied to test for significant differences in stomatal response to ABA treatment. More than 400 stomata were measured in each group, and at least 10 stomata were measured on each frond. GO terms and KEGG pathways that were statistically significantly enriched in the expanded gene families were tested using OmicShare Tools (http://www.omicshare.com/tools) by conducting a hypergeometric test.

## Supplementary Material

qzaf074_Supplementary_Data

## Data Availability

The raw whole-genome sequencing data of *L*. *punctata* strain 0202 generated in this study have been deposited in the NCBI (BioProject ID: PRJNA546087) and the Genome Sequence Archive [103] at the National Genomics Data Center (NGDC) [104], China National Center for Bioinformation (CNCB) (GSA: CRA017759) that are publicly accessible at https://ngdc.cncb.ac.cn/gsa. The raw RNA-seq data have been deposited in the NCBI (BioProject ID: PRJNA670783, PRJNA670784, and PRJNA670786) and the GSA [103] at the NGDC [104], CNCB (GSA: CRA017718) that are publicly accessible at https://ngdc.cncb.ac.cn/gsa.

## CRediT author statement

**Yang Fang:** Formal analysis, Funding acquisition, Investigation, Project administration, Supervision, Validation, Writing – original draft, Writing – review & editing. **Xueping Tian:** Data curation, Investigation, Methodology, Software, Validation, Visualization, Writing – original draft, Writing – review & editing. **Yanling Jin:** Funding acquisition, Methodology, Project administration, Supervision, Validation, Writing – review & editing. **Anping Du:** Data curation, Validation, Visualization, Writing – original draft, Writing – review & editing. **Yanqiang Ding:** Data curation, Formal analysis, Investigation, Methodology, Software, Validation, Visualization, Writing – original draft, Writing – review & editing. **Zhihua Liao:** Formal analysis, Writing – review & editing. **Kaize He:** Formal analysis, Investigation, Writing – review & editing. **Yonggui Zhao:** Writing – review & editing. **Ling Guo:** Data curation, Methodology, Validation, Visualization, Writing – original draft, Writing – review & editing. **Yao Xiao:** Data curation, Methodology, Visualization. **Yaliang Xu:** Data curation, Methodology, Validation, Writing – original draft, Writing – review & editing. **Shuang Chen:** Validation, Writing – original draft, Writing – review & editing. **Yuqing Che:** Data curation, Methodology, Software, Validation, Visualization. **Li Tan:** Data curation, Software, Validation, Writing – original draft. **Songhu Wang:** Formal analysis, Writing – original draft, Writing – review & editing. **Jiatang Li:** Visualization, Writing – review & editing. **Zhuolin Yi:** Investigation, Methodology, Validation, Writing – review & editing. **Lanchai Chen:** Investigation, Writing – review & editing. **Leyi Zhao:** Writing – review & editing. **Fangyuan Zhang:** Data curation, Writing – review & editing. **Guoyou Li:** Data curation, Writing – review & editing. **Jinmeng Li:** Validation, Writing – review & editing. **Qinli Xiong:** Writing – review & editing. **Yongmei Zhang:** Writing – review & editing. **Qing Zhang:** Writing – review & editing. **Xuan Hieu Cao:** Writing – review & editing. **Hai Zhao:** Conceptualization, Formal analysis, Funding acquisition, Investigation, Methodology, Project administration, Resources, Supervision, Writing – original draft, Writing – review & editing. All authors have read and approved the final manuscript.

## ORCID

0000-0002-4535-457X (Yang Fang)

0000-0002-9556-2588 (Xueping Tian)

0000-0001-7667-8115 (Yanling Jin)

0000-0001-8697-7725 (Anping Du)

0000-0002-7740-6696 (Yanqiang Ding)

0000-0002-1283-4649 (Zhihua Liao)

0000-0001-5077-7385 (Kaize He)

0000-0002-6630-1717 (Yonggui Zhao)

0000-0003-1986-3175 (Ling Guo)

0000-0001-5727-4609 (Yao Xiao)

0000-0002-9577-1726 (Yaliang Xu)

0009-0000-2805-4797 (Shuang Chen)

0009-0005-4894-8751 (Yuqing Che)

0000-0001-5993-8544 (Li Tan)

0000-0002-7822-769X (Songhu Wang)

0000-0003-1799-194X (Jiatang Li)

0009-0005-6877-2861 (Zhuolin Yi)

0000-0001-6681-1509 (Lanchai Chen)

0009-0003-2460-0916 (Leyi Zhao)

0000-0003-3840-9947 (Fangyuan Zhang)

0000-0001-9690-4044 (Guoyou Li)

0000-0002-1619-3973 (Jinmeng Li)

0000-0003-3827-3437 (Qinli Xiong)

0000-0002-7777-0420 (Yongmei Zhang)

0000-0002-2940-7440 (Qing Zhang)

0000-0003-1230-4127 (Xuan Hieu Cao)

0000-0002-6116-6149 (Hai Zhao)
