# Supplementary material for: Duckweed Evolution: from Land back to Water
Source: Genomics Proteomics Bioinformatics. 2025 Aug 23;23(4):qzaf074. doi: 10.1093/gpbjnl/qzaf074 (PMC12707978; doi:10.1093/gpbjnl/qzaf074)
Supplement: qzaf074_Supplementary_Data [file qzaf074_supplementary_data.zip › File_S1.docx]

**File S1 The genomic information of *Landolina punctata* strain 0202**

**Chromosomes of *Landolina punctata* strain 0202**

Metaphase spreads indicated that *L*. *punctata* strain 0202 showed 2n = 40 for chromosome number (Figure S13), the same as *Spirodela polyrhiza* strain 7498 and *Lemna minor* strain 5500. However, this result was different from the previous study with chromosome number of 2n = 46, which reported no chromosome number variation between the tested clones of *Spirodela* and *Landoltia* species [1]. This discrepancy might arise from methodological sensitivity or biological variation among strains. Since the intra-specific and inter-specific karyotype variation exists commonly in duckweeds [2], systematic analysis of additional clones is essential to investigate the presence and mechanisms of chromosome number variation within the Lemnaceae family.

**Estimation of the genome size of *L. punctata*** **strain 0202**

Sequencing generated 166.4 Gb of raw reads. After removing adapter sequences, low-quality reads, and duplicate reads, 96.6 Gb of clean reads remained (Table S22). More than 80% of bases had sequencing depths of > 100×. Bases with sequencing depth of < 10× accounted for approximately 2.4%. The average sequencing depth was 224.4× (Figure S14). The sequencing depth was sufficient for subsequent genome assembly (Table S23). The estimated genome size of *L. punctata* strain 0202 was approximately 415 Mb (Figure S15; Table S24), which was larger than the genome size of *L. punctata* clone 5635 (350 Mb with 60× coverage) [3].

**Chromosome anchoring**

Figure S16 shows that gene homology is conserved between the two species. In total, 403.5 Mb (95.5%) of the sequences of *L. punctata* anchored to chromosomes. The remaining scaffolds containing approximately 18.9 Mb were combined as chromosome 0.

**Summary of assembly and annotation statistics of *L. punctata* strain 0202**

The final assembly consisted of 48,966 scaffolds (63,986 contigs), covering 422.4 Mb of the genome with a scaffold L50 of 4.0 Mb and corresponding contig L50 of 54.0 kb; these summaries were longer than most of plant assemblies applying next generation sequencing (Tables S25 and S26). The GC content of *L. punctata* strain 0202 is 36.5% (Table S25).

Analyses predicted 19,692 protein-coding genes in *L. punctata* strain 0202 whose length averaged 3586 bp, with an average of 5 exons. The functions of 15,890 predicted genes (80.7%) were identified (Tables S27 and S28). Repetitive sequences accounted for 59.2% of the genome (Table S29), which was higher than that of *S. polyrhiza* (*S. polyrhiza* strain 7498, 14.7%; *S. polyrhiza* strain 9509, ~ 25.3%). In the genome of *L. punctata*, long terminal repeats (LTRs) were the most abundant retrotransposon, accounting for 19.4% of the assembly (Table S30).

Quality of assembled genome and annotation was assessed by Benchmarking Universal Single-Copy Orthologs (BUSCO) and/or Expressed Sequence Tags (EST). For the genome assembly evaluation, we compared the genome assembly quality of *L. punctata* strain 0202 with those of the model plants and species related to duckweed. BUSCO assessment, including 303 BUSCOs, showed that 88.8% of the set of core eukaryotic genes were present (248 complete single-copy and 21 complete duplicated). The numbers of fragmented and missing BUSCOs were 7 and 27, respectively. Our assembly had more completely represented BUSCOs than all of the model plants, except *Arabidopsis* (Figure S17). EST assessment showed that 3720 ESTs out of 3794 (98.0%) were mapped to the assembly (Tables S31 and S32). This indicated a high quality of genome assembly. For genome annotation evaluation, among 303 BUSCOs, 95.1% of the set of core eukaryotic genes were present completely (261 complete single-copy and 27 completely duplicated). The numbers of fragmented and missing BUSCOs were 11 and 4, respectively. Compared with the assessments of relative species, the most BUSCOs of *L. punctata* strain 0202 were detected completely, indicating high quality of annotation in this study (Figure S18).

**References**

[1] Hoang PTN, Schubert V, Meister A, Fuchs J, Schubert I. Variation in genome size, cell and nucleus volume, chromosome number and rDNA loci among duckweeds. Sci Rep 2019;9:3234.

[2] Cao XH, Vu GTH. Cytogenetics, epigenetics and karyotype evolution of duckweeds. In: Cao XH, Fourounjian P, Wang W, editors. The duckweed genomes. Cham: Springer; 2020, p.47–57.

[3] Baggs EL, Tiersma MB, Abramson BW, Michael TP, Krasileva KV. Characterization of defense responses against bacterial pathogens in duckweeds lacking EDS1. New Phytol 2022;236:1838–55.
