# Supplementary material for: Duckweed Evolution: from Land back to Water
Source: Genomics Proteomics Bioinformatics. 2025 Aug 23;23(4):qzaf074. doi: 10.1093/gpbjnl/qzaf074 (PMC12707978; doi:10.1093/gpbjnl/qzaf074)
Supplement: qzaf074_Supplementary_Data [file qzaf074_supplementary_data.zip › Supplementary Material Captions.docx]

**File S1 The genomic information of *Landolina punctata* strain 0202**

**Figure S1 Phylogenetic analysis based on chloroplast genomes from algae (blue), terrestrial plants (yellow), and aquatic plants (green)**

Numbers at the nodes are bootstrap values. Tree was rooted using *Chlamydomonas reinhardtii* as the outgroup taxon. The eudicots included *Glycine max*, *Medicago truncatula*, *Carica papaya*, *Arabidopsis thaliana*, *Vitis vinifera*, and *Populus trichocarpa*. ★, the ancestor of duckweeds (*Limnobiophyllum scutatum*).

**Figure S2 The fossil reconstruction of *Limnobiophyllum scutatum***

Scale bar, 2 cm.

**Figure S3 Expression levels of genes involved in root development in duckweeds**

Numbers in the boxes are FPKM values. The black boxes mean genes negatively related to root development. NA, genes lost. Detailed gene names are provided in Table S2.

**Figure S4 Expression levels of genes involved in stomata development in duckweeds**

Numbers in the boxes are FPKM values. The black boxes mean genes not related to stomata development. NA, genes lost. Detailed gene names are provided in Table S4.

**Figure S5 Detection of phytohormones in duckweeds**

IAA, indole-3-acetic acid; ME-IAA, methylindole-3-acetic acid; IBA, indole-3-butyric acid; ICA, indole-3-carboxaldehyde; IP, isopentenyl adenine; tZ, trans-Zeatin; cZ, cis-Zeatin; DZ, dihydro-zeatin; H2JA, dihydro jasmonic acid; JA-ILE, jasmonic acid-isoleucine; GA3, gibberellin 3; GA4, gibberellin 4.

**Figure S6 Distribution of stomatal aperture in duckweeds under ABA treatment**

Duckweeds were placed in 1/5 Hoagland for 3 h under white light to induce stomata opening and further incubated in the absence (control groups) or in the presence 100 μM ABA (ABA groups).

**Figure S7 Phylogenetic analyses of LAC genes in *Arabidopsis thaliana* and duckweeds**

Key genes involved in lignin synthesis of *Arabidopsis thaliana* (*AtLAC4*, *AtLAC11*, and *AtLAC17*) were highlighted in red. One homologous gene of *AtLAC11* occurs in each duckweed genome, one *AtLAC17* homologue in *Spirodela polyrhiza*, and no *AtLAC4* homologues in duckweeds. LAC, laccase.

**Figure S8 Numbers of LEA protein genes in duckweeds and terrestrial plants**

Kfl, *Klebsormidium flaccidum*; Zos, *Zostera marina*; Ath, *Arabidopsis thaliana*; Osa, *Oryza sativa*; Zma, *Zea mays*. Detailed gene information provided in Table S12. LEA, late embryogenesis abundant.

**Figure S9 Numbers of TF genes involved in stress response in duckweeds and land plants**

**A.** Number of TF genes in the families bHLH, C2H2, and WRKY. Detailed gene information was provided in Table S14C, D, and E.**B.** Number of TF genes involved in the drought stress signaling network. Detailed gene information was provided in Table S14A. TF, transcription factor.

**Figure S10 Enriched top 30 KEGG pathways of expanded gene families in duckweeds as compared with model plants *Arabidopsis thaliana*, *Oryza sativa*, and *Zea mays***

**A.** Enriched top 30 pathways in *Spirodela polyrhiza*. **B.** Enriched top 30 pathways in *Landoltia punctata*. **C.** Enriched top 30 pathways in *Lemna minor*. Detail information provided in Table S15. The flavonoid, anthocyanin, flavone, and flavonol biosynthesis pathways are enriched in duckweeds, which is consistent with the high flavonoid content of duckweeds. KEGG, Kyoto Encyclopedia of Genes and Genomes.

**Figure S11 The content and variety of flavonoids in duckweeds**

**A.** The content of total flavonoids in duckweeds. **B.** The content of anthocyanins in duckweeds. **C.** The HPLC-MS and HPLC-TIC chromatograms of *Landoltia punctata* in negative ion mode. Upper panel, middle panel, and lower panel show the HPLC-TIC, HPLC-MS, and HPLC chromatograms, respectively. There are more than 30 obvious peaks observed in both HPLC-MS and HPLC-TIC chromatograms, indicating more than 30 flavonoids in *Landoltia punctata*. HPLC, high performance liquid chromatography; MS, mass spectrometry; TIC, total ion chromatogram.

**Figure S12 The comparison of SnRK2 (III) coding sequence in the reference genome of *Spirodela polyrhiza*, *Landoltia punctata*, *Lemna minor*, and *Lemna minor***

Spipo3G0113400_SnRK2 (III) is the Group III SnRK2 in *Spirodela polyrhiza*, Landoltia_punctata_GLEAN_10003251_SnRK2 (III) is the Group III SnRk2 in *Landoltia punctata*, Lemna minor 7753_SnRK2 (III) is the Group III SnRK2 in *Lemna minor*, and Lminor_002649.mRNA1 is suspected Group III SnRK2 in *Lemna minor*.

**Figure S13 Metaphase chromosomes of *Landoltia punctata* (2n = 40)**

**Figure S14 Genomic sequencing depth**

**Figure S15 K-mer frequency distribution based on a 17-mer**

**Figure S16 Syntenic circos plot of genomic base-pairs of *Landoltia punctata* strain 0202 and *Spirodela polyrhiza* strain 9509**

The “L” and “S” associating with a number present the chromosome of *Landoltia punctata* and *Spirodela polyrhiza*, respectively.

**Figure S17 Assessment of genome assembly using BUSCO**

Aco, *Ananas comosus* (L.); Atr, *Amborella trichopoda*; Cbr, *Chara braunii*; Mpo, *Marchantia polymorpha*; Nnu, *Nelumbo nucifera*; Spo7498, *Spirodela polyrhiza* strain 7498; Spo9509, *Spirodela polyrhiza* strain 9509; Van, *Vigna angularis*; BUCSO, Benchmarking Universal Single-Copy Orthologs. .

**Figure S18 Assessment of genome annotation using BUSCO**

**Table S1 Data links of genomes used in this study**

**Table S2 Detailed information and expression level of genes involved in root development**

**Table S3 The gene number and gene expression of auxin signaling pathway involved in AR development**

**Table S4 Detailed information and expression level of genes involved in stomata development**

**Table S5 Number of genes involved in phytohormone pathways**

**Table S6 List of genes involved in the SCF core signaling pathway of auxin, jasmonic acid, and gibberellic acid**

**Table S7 Gene number of main components involved in ABA-induced stomatal closure signaling in eight typical species**

**Table S8 The content of cellulose, hemicellulose, and lignin of *C*. *reinhardtii*, duckweeds, and other plants**

**Table S9 Number and expression level of genes involved in lignin biosynthesis**

**Table S10 Number and expression level of genes involved in cellulose biosynthesis**

**Table S11 Number and expression level of genes involved in hemicellulose biosynthesis**

**Table S12 Detail information and number of LEA protein genes**

**Table S13 Total number of TFs in duckweeds predicted by iTAK**

**Table S14 Detail information and number of TFs in bHLH, C2H2, and WRKY family**

**Table S15 The list of expanded gene families enriched in flavonoid, anthocyanin, flavone, and flavonol biosynthesis pathways**

**Table S16 The number of genes involved in phenylalanine metabolic pathway**

**Table S17 The expanded gene families list of PPR proteins**

**Table S18 The number of TEs in three duckweed species**

**Table S19 Detail information about GO enrichment analysis of expanded gene families in duckweeds**

**Table S20 Detail information about KEGG enrichment analysis of expanded gene families in duckweeds**

**Table S21 The Ka/Ks analysis results between land plants and duckweeds**

**Table S22 Statistics of raw and filtered genomic sequencing data**

*Note*: PE, paired-end libraries; MP, mate-pair libraries.

**Table S23** **The size and gene number of published plants genomes**

**Table S24 Result of 17-mer frequency distribution analyses**

**Table S25 Summary of assembly statistics of *Landoltia punctata***

**Table S26 Comparison of assembly quality between *Landoltia punctata* and other plants with published genome**

*Note*: -, data not available.

**Table S27 Statistics of predicted protein-coding genes in the *Landoltia punctata***

*Note*: GN, gene number; AGL, average gene length; ACL, average CDA length; TEx, total exon; AExN, average exon number; AExL, average exon length; AInL, average intron length; CDA, coding DNA; EST, expressed sequence tags.

**Table S28 Number of genes with homology or functional classifications by different methods**

**Table S29 Repetitive sequences annotation in the assembly of *Landoltia punctata***

**Table S30 Types of TEs in *Landoltia punctata***

**Table S31 Assessment of genome assembly using expressed sequence tags**

**Table S32 Summary of assessment of genome assembly with expressed sequence tags**

**Table S33 Characteristics of RNA-sequencing data**

**Table S34 The results of clean reads alignment with rRNA**
