# Supplementary material for: Duckweed Evolution: from Land back to Water
Source: Genomics Proteomics Bioinformatics. 2025 Aug 23;23(4):qzaf074. doi: 10.1093/gpbjnl/qzaf074 (PMC12707978; doi:10.1093/gpbjnl/qzaf074)
Supplement: qzaf074_Supplementary_Data [file qzaf074_supplementary_data.zip › Table_S1.docx]

Table S1 Data links of genomes used in this study

| **Species** | **Links** |
| --- | --- |
| *Ostreococcus lucimarinus* | https://www.ncbi.nlm.nih.gov/datasets/genome/GCF_000092065.1/ |
| *Klebsormidium flaccidum* | http://www.plantmorphogenesis.bio.titech.ac.jp/~algae_genome_project/klebsormidium/kf_download.htm |
| *Chlamydomonas reinhardtii* | https://www.ncbi.nlm.nih.gov/datasets/genome/GCF_000002595.1/ |
| *Marchantia polymorpha* | https://www.ncbi.nlm.nih.gov/datasets/genome/GCA_001641455.1/ |
| *Spirodela polyrhiza* | https://www.ncbi.nlm.nih.gov/datasets/genome/GCA_001981405.1/ |
| *Landoltia punctata* | https://ngdc.cncb.ac.cn/gsub/submit/bioproject/subPRO041525/overview |
| *Zostera marina* | https://www.ncbi.nlm.nih.gov/datasets/genome/GCA_001185155.1/ |
| *Selaginella moellendorffii* | https://www.ncbi.nlm.nih.gov/datasets/genome/GCF_000143415.3/ |
| *Lemna minor* | https://genomevolution.org/coge/SearchResults.pl?s=27419&p=genome |
| *Nelumbo nucifera Gaertn* | https://www.ncbi.nlm.nih.gov/datasets/genome/GCF_000365185.1/ |
| *Amborella trichopoda* | https://www.ncbi.nlm.nih.gov/datasets/genome/GCF_000471905.2/ |
| *Arabidopsis thaliana* | https://www.ncbi.nlm.nih.gov/datasets/genome/GCF_000001735.3/ |
| *Physcomitrella pattens* | https://www.ncbi.nlm.nih.gov/datasets/genome/GCF_000002425.3/ |
| *Picea abies* | https://www.omicsdi.org/dataset/project/PRJEB1822 |
| *Utricularia gibba* | https://www.ncbi.nlm.nih.gov/datasets/genome/GCA_002189035.1/ |
| *Phyllostachys heterocycla* | https://www.ebi.ac.uk/ena/browser/view/ERP001340&display |
| *Eucalyptus grandis* | https://www.ncbi.nlm.nih.gov/datasets/genome/GCF_000612305.1/ |
| *Oryza sativa* | https://www.ncbi.nlm.nih.gov/datasets/genome/GCF_000005425.2/ |
| *Zea mays ssp. mays* | https://www.ncbi.nlm.nih.gov/datasets/genome/GCF_000005005.2/ |
| *Hevea brasiliensis* | https://www.ncbi.nlm.nih.gov/datasets/genome/GCF_001654055.1/ |
| *Camellia sinensis* | https://www.ncbi.nlm.nih.gov/bioproject/PRJNA381277/ |
| *Populus trichocarpa* | https://www.ncbi.nlm.nih.gov/datasets/genome/GCF_000002775.3/ |
| *Apostasia shenzhenica* | https://www.ncbi.nlm.nih.gov/datasets/genome/GCA_002786265.1/ |
