# Supplementary material for: Duckweed Evolution: from Land back to Water
Source: Genomics Proteomics Bioinformatics. 2025 Aug 23;23(4):qzaf074. doi: 10.1093/gpbjnl/qzaf074 (PMC12707978; doi:10.1093/gpbjnl/qzaf074)
Supplement: qzaf074_Supplementary_Data [file qzaf074_supplementary_data.zip › Table_S18.docx]

Table S18 The number of TEs in three duckweed species

| Type | Spo (%) | Lpu (%) | Lmi (%) |
| --- | --- | --- | --- |
| DNA transposons | NA | 16 | 5.08 |
| Retrotransposon | 13.06 | 21.9 | 31.2 |
| Other | 1.66 | NA | 3.91 |
| Unknown | NA | 21.2 | 21.27 |
| Total | 14.72 | 52.50 | 61.46 |

*Note*: TE, transposable element.
