# Supplementary material for: Duckweed Evolution: from Land back to Water
Source: Genomics Proteomics Bioinformatics. 2025 Aug 23;23(4):qzaf074. doi: 10.1093/gpbjnl/qzaf074 (PMC12707978; doi:10.1093/gpbjnl/qzaf074)
Supplement: qzaf074_Supplementary_Data [file qzaf074_supplementary_data.zip › Table_S22.docx]

**Table S22 Statistics of raw and filtered genomic sequencing data**

| **Library** | **Insert size (bp)** | **Library type** | **Read length (bp)** | **Raw data (Gb)** | **Clean data (Gb)** | **Read Q20 (read1; read2) (%)** | **Coverage (×)** |
| --- | --- | --- | --- | --- | --- | --- | --- |
| wHAXPI000147-26 | 200 | PE | 100 | 19.0 | 16.2 | 99.8; 99.7 | 80.6 |
| wHAXPI009506-104 | 200 | PE | 100 | 19.8 | 18.4 | 99.7; 99.6 |  |
| wHAIPI000148-97 | 500 | PE | 100 | 16.1 | 12.0 | 99.8; 98.5 | 55.6 |
| wHAIPI009496-105 | 500 | PE | 100 | 14.1 | 11.9 | 99.8; 98.0 |  |
| wHAMPI012249-33 | 800 | PE | 100 | 13.2 | 11.7 | 99.8; 98.5 | 51.8 |
| wHAMPI012250-33 | 800 | PE | 100 | 13.0 | 10.6 | 99.8; 95.9 |  |
| WHLEMdamDEAADWAAPEI-30 | 2000 | MP | 90 | 8.7 | 4.8 | 99.8; 98.6 | 11.1 |
| WHLEMdamDEABDLAAPEI-31 | 5000 | MP | 90 | 8.1 | 3.6 | 99.8; 98.2 | 8.4 |
| WHLEMdamDGAADTAAPEI-11 | 10,000 | MP | 50 | 15.1 | 2.0 | 99.7; 92.7 | 4.6 |
| WHLEMdamDHAADUAAPEI-8 | 20,000 | MP | 50 | 18.0 | 2.6 | 99.9; 97.1 | 12.4 |
| WHLEMdamDHABDUBAPEI-9 | 20,000 | MP | 50 | 21.3 | 2.8 | 99.8; 97.1 |  |
| Total | / | / | / | 166.4 | 96.6 | / | 224.4 |

*Note*: PE, paired-end libraries; MP, mate-pair libraries.
