# Supplementary material for: Duckweed Evolution: from Land back to Water
Source: Genomics Proteomics Bioinformatics. 2025 Aug 23;23(4):qzaf074. doi: 10.1093/gpbjnl/qzaf074 (PMC12707978; doi:10.1093/gpbjnl/qzaf074)
Supplement: qzaf074_Supplementary_Data [file qzaf074_supplementary_data.zip › Table_S23.docx]

**Table S23 The size and gene number of published plants genomes**

| **Species** | **Size** | **Gene number** | **Species** | **Size** | **Gene number** |
| --- | --- | --- | --- | --- | --- |
| *Klebsormidium flaccidum* | 104 Mb | 16,215 | *Picea abies* | 19.6 Gb | 28,354 |
| *Saccharina japonica* | 537 Mb | 18,733 | *Oropetium thomaeum* | 245 Mb | 28,466 |
| *Zostera marina* | 202 Mb | 20,450 | *Phalaenopsis equestris* | 1.086 Gb | 29,431 |
| *Spirodela polyrhiza* | 158 Mb | 19,623 | *Salvia miltiorrhiza* | 538 Mb | 30,478 |
| *Landoltia punctata* | 423 Mb | 19,692 | *Phaseolus vulgaris* L. | 549 Mb | 30,491 |
| *Lemna minor* | 472 Mb | 22,382 | *Phyllostachys heterocycla* | 2.05 Gb | 31,987 |
| *Selaginella moellendorffii* | 212 Mb | 22,285 | *Vigna angularis* | 466 Mb | 34,183 |
| *Sesamum indicum* L. | 293 Mb | 23,713 | *Elaeis guineensis* | 1.535 Gb | 34,802 |
| *Setaria italica* | 400 Mb | 24,000 | *Capsicum annuum* | 3.06 Gb | 34,903 |
| *Coffea canephora* | 568 Mb | 25,574 | *Hordeum vulgare* L. var. *nudum* | 3.89 Gb | 36,151 |
| *Nelumbo nucifera Gaertn* | 804 Mb | 26,685 | *Eucalyptus grandis* | 605 Mb | 36,376 |
| *Amborella trichopoda* | 706 Mb | 26,846 | *Musa acuminata* | 472 Mb | 36,542 |
| *Ananas comosus* (L.) Merr. | 382 Mb | 27,024 | *Oryza sativa* | 466 Mb | 39,049 |
| *Arabidopsis thaliana* | 125 Mb | 27,416 | *Zea mays* ssp. *mays* | 2.3 Gb | 39,190 |
| *Beta vulgaris* | 566 Mb | 27,421 | *Hevea brasiliensis* | 1.37 Gb | 43,792 |
| *Physcomitrella pattens* | 480 Mb | 27,970 |  |  |  |
