# Supplementary material for: Duckweed Evolution: from Land back to Water
Source: Genomics Proteomics Bioinformatics. 2025 Aug 23;23(4):qzaf074. doi: 10.1093/gpbjnl/qzaf074 (PMC12707978; doi:10.1093/gpbjnl/qzaf074)
Supplement: qzaf074_Supplementary_Data [file qzaf074_supplementary_data.zip › Table_S24.docx]

**Table S24 Result of 17-mer frequency distribution analyses**

| **Insert size (bp)** | **K-mer number** | **Peak depth** | **Estimated genome size** |
| --- | --- | --- | --- |
| 200 | 29,071,396,956 | 70 | 415,305,670 |
| 500 | 20,058,033,744 | 48 | 417,875,703 |
| 800 | 18,671,762,844 | 45 | 414,928,063 |
