# Supplementary material for: Duckweed Evolution: from Land back to Water
Source: Genomics Proteomics Bioinformatics. 2025 Aug 23;23(4):qzaf074. doi: 10.1093/gpbjnl/qzaf074 (PMC12707978; doi:10.1093/gpbjnl/qzaf074)
Supplement: qzaf074_Supplementary_Data [file qzaf074_supplementary_data.zip › Table_S25.docx]

**Table S25 Summary of assembly statistics of *Landoltia punctata***

| **/** | **Contigs** | |  | **Scaffolds** | |
| --- | --- | --- | --- | --- | --- |
|  | **Length (bp)** | **Number** |  | **Length (bp)** | **Number** |
| L50 | 54,011 | 2164 |  | 4,030,252 | 30 |
| Max length | 1,111,587 | - |  | 17,897,791 | - |
| Number (≥ 100 bp) | - | 63,986 |  | - | 48,966 |
| Number (≥ 2000 bp) | - | 12,630 |  | - | 745 |
| Total size | 403,354,422 | - |  | 422,361,459 | - |
| GC content | 39.1% | |  | 36.5% | |
