# Supplementary material for: Duckweed Evolution: from Land back to Water
Source: Genomics Proteomics Bioinformatics. 2025 Aug 23;23(4):qzaf074. doi: 10.1093/gpbjnl/qzaf074 (PMC12707978; doi:10.1093/gpbjnl/qzaf074)
Supplement: qzaf074_Supplementary_Data [file qzaf074_supplementary_data.zip › Table_S26.docx]

**Table S26 Comparison of assembly quality between *Landoltia punctata* and other plants with published genome**

| **Species** | **Contig L50** | **Scaffold L50** | **Genome size**  **assembly** |
| --- | --- | --- | --- |
| *Landoltia punctata* | 54.0 kb | 4.0 Mb | 422.4 Mb |
| *Selaginella moellendorffii* | 119.8 kb | 1.7 Mb | 212.6 Mb |
| *Musa acuminata* | - | 1.3 Mb | 472.2 Mb |
| *Oryza sativa* L. ssp. *indica* | 6.69 kb | 11.76 kb | 466 Mb |
| *Sesamum indicum* L. | 52.2 kb | 2.1 Mb | 274 Mb |
| *Phyllostachys heterocycla* | 11.8 kb | 328 kb | 2.05 Gb |
| *Nelumbo nucifera* Gaertn. | 38.8 kb | 3.4 Mb | 804 Mb |
| *Elaeis guineensis* | - | 1.27 Mb | 1.535 Gb |
| *Amborella trichopoda* | - | 4.9 Mb | 706 Mb |
| *Capsicum annuum* | - | 2.47 Mb | 3.06 Gb |
| *Beta vulgaris* | - | 2.01 Mb | 566.6 Mb |
| *Spirodela polyrhiza* | - | 3.7 Mb | 145 Mb |
| *Eucalyptus grandis* | 2.3 Mb | 5 Mb | 605 Mb |
| *Coffea canephora* | - | 1.26 Mb | 568.6 Mb |
| *Phalaenopsis equestris* | 20.5 kb | 359 kb | 1.086 Gb |
| *Hordeum vulgare* L. var. *nudum* | 18.1 kb | 242 kb | 3.89 Gb |
| *Saccharina japonica* | 58.8 kb | 252 kb | 537 Mb |
| *Ananas comosus* (L.) Merr. | 126.5 kb | 11.8 Mb | 382 Mb |
| *Lemna minor* | 20.9 kb | 23.6 kb | 472Mb |
| *Oropetium thomaeum* | 2.4 Mb | - | 245 Mb |
| *Zostera marina* | 79.9 kb | 485.5 kb | 202.3 Mb |
| *Phaseolus vulgaris* L. | 3.27 Mb | 5 Mb | 587 Mb |
| *Salvia miltiorrhiza* | 12.38 kb | 51.02 kb | 538 Mb |
| *Hevea brasiliensis* | **-** | 1.28 Mb | 1.37 Gb |

*Note*: -, data not available.
