# Supplementary material for: Duckweed Evolution: from Land back to Water
Source: Genomics Proteomics Bioinformatics. 2025 Aug 23;23(4):qzaf074. doi: 10.1093/gpbjnl/qzaf074 (PMC12707978; doi:10.1093/gpbjnl/qzaf074)
Supplement: qzaf074_Supplementary_Data [file qzaf074_supplementary_data.zip › Table_S27.docx]

**Table S31 Statistics of predicted protein-coding genes in the *Landoltia punctata***

| **Gene set** | | **GN** | **AGL (bp)** | **ACL (bp)** | **TEx** | **AExN** | **AExL (bp)** | **AInL (bp)** |
| --- | --- | --- | --- | --- | --- | --- | --- | --- |
| Homolog search | |  |  |  |  |  |  |  |
|  | *Arabidopsis thaliana* | 14,057 | 3138.2 | 1140.4 | 72,644 | 5.2 | 220.7 | 479.3 |
|  | *Oryza sativa* | 14,830 | 3118.3 | 1132.4 | 72,281 | 4.9 | 232.3 | 512.6 |
|  | *Zea mays* | 14,334 | 2807.4 | 1079.4 | 69,433 | 4.8 | 222.8 | 449.5 |
|  | *Brachypodium distachyon* | 13,363 | 3089.1 | 1131.7 | 69,075 | 5.2 | 218.9 | 469.5 |
|  | *Sorghum bicolor* | 14,625 | 2888.9 | 1085.2 | 71,494 | 4.9 | 222.0 | 463.9 |
|  | *Spirodela polyrhiza* | 16,174 | 2923.3 | 1085.8 | 77,044 | 4.8 | 228.0 | 488.2 |
|  | *Lemna minor* | 15,849 | 3116.4 | 1135.4 | 79,486 | 5.0 | 226.4 | 493.4 |
| *De novo* | |  |  |  |  |  |  |  |
|  | AUGUSTUS | 32,896 | 3051.1 | 968.1 | 129,643 | 3.9 | 245.6 | 708.3 |
|  | GENSCAN | 50,098 | 5503.7 | 1027.4 | 264,326 | 5.3 | 194.7 | 1046.8 |
| RNA | |  |  |  |  |  |  |  |
|  | expressed sequence tags (EST) | 982 | 5929.6 | 707.4 | 3764 | 3.8 | 184.6 | 1843.3 |
|  | Transcripts | 24,735 | 7720.2 | 2640.0 | 217,825 | 8.8 | 299.8 | 650.8 |
| Total gene set | | 19,692 | 3585.9 | 1142.9 | 99,846 | 5.1 | 225.4 | 600.2 |

*Note*: GN, gene number; AGL, average gene length; ACL, average coding DNA (CDA) length; TEx, total exon; AExN, average exon number; AExL, average exon length; AInL, average intron length.
