# Supplementary material for: Duckweed Evolution: from Land back to Water
Source: Genomics Proteomics Bioinformatics. 2025 Aug 23;23(4):qzaf074. doi: 10.1093/gpbjnl/qzaf074 (PMC12707978; doi:10.1093/gpbjnl/qzaf074)
Supplement: qzaf074_Supplementary_Data [file qzaf074_supplementary_data.zip › Table_S28.docx]

**Table S32 Number of genes with homology or functional classifications by different methods**

|  | **Annotated number** | **Percent (%)** |
| --- | --- | --- |
| InterPro | 15,176 | 77.1 |
| GO | 11,764 | 59.7 |
| KEGG | 11,633 | 59.1 |
| Swiss-Prot | 11,857 | 60.2 |
| Total annotated | 15,890 | 80.7 |
