# Supplementary material for: Duckweed Evolution: from Land back to Water
Source: Genomics Proteomics Bioinformatics. 2025 Aug 23;23(4):qzaf074. doi: 10.1093/gpbjnl/qzaf074 (PMC12707978; doi:10.1093/gpbjnl/qzaf074)
Supplement: qzaf074_Supplementary_Data [file qzaf074_supplementary_data.zip › Table_S29.docx]

**Table S29 Repetitive sequences annotation in the assembly of *Landoltia punctata***

| **Methods** | | **Repeat size (bp)** | **Percent (%)** |
| --- | --- | --- | --- |
| Homology-based | |  |  |
|  | TRF | 54,373,159 | 12.9 |
|  | RepeatMasker | 40,126,737 | 9.5 |
|  | RepeatProteinMask | 33,969,413 | 8.0 |
| *De novo* | |  |  |
|  | RepeatModeler | 218,847,027 | 51.8 |
| Total | | 250,178,678 | 59.2 |

*Note*: TRF, Tandem Repeats Finder.
