# Supplementary material for: Duckweed Evolution: from Land back to Water
Source: Genomics Proteomics Bioinformatics. 2025 Aug 23;23(4):qzaf074. doi: 10.1093/gpbjnl/qzaf074 (PMC12707978; doi:10.1093/gpbjnl/qzaf074)
Supplement: qzaf074_Supplementary_Data [file qzaf074_supplementary_data.zip › Table_S30.docx]

**Table S30 Types of TEs in *Landoltia punctata***

| **Type** | | **Length (bp)** | **Percent (%)** |
| --- | --- | --- | --- |
| DNA transposons | | 67,513,324 | 16.0 |
| Retrotransposon | |  |  |
|  | LINE | 10,390,636 | 2.5 |
|  | SINE | 162,159 | 0.0 |
|  | LTR | 81,862,377 | 19.4 |
|  | Other | 3698 | 0.0 |
| Unknown | | 89,547,421 | 21.2 |
| Total | | 221,800,767 | 52.5 |
