# Supplementary material for: Duckweed Evolution: from Land back to Water
Source: Genomics Proteomics Bioinformatics. 2025 Aug 23;23(4):qzaf074. doi: 10.1093/gpbjnl/qzaf074 (PMC12707978; doi:10.1093/gpbjnl/qzaf074)
Supplement: qzaf074_Supplementary_Data [file qzaf074_supplementary_data.zip › Table_S31.docx]

**Table S27 Assessment of genome assembly using expressed sequence tags**

| **Dataset** | **Total number** | **Total length** | **Covered by assembly (%)** | **With > 90% sequence in one scaffold** | |  | **With > 50% sequence in one scaffold** | |
| --- | --- | --- | --- | --- | --- | --- | --- | --- |
|  |  |  |  | **Number** | **Ratio** |  | **Number** | **Ratio** |
| ≥ 0 bp | 3794 | 2,397,521 | 93.8 | 2950 | 77.8 |  | 3720 | 98.1 |
| ≥ 200 bp | 3794 | 2,397,521 | 93.8 | 2950 | 77.8 |  | 3720 | 98.1 |
| ≥ 500 bp | 2862 | 2,040,428 | 94.8 | 2559 | 89.4 |  | 2806 | 98.0 |
| ≥ 1000 bp | 138 | 144,166 | 95.4 | 132 | 95.7 |  | 133 | 96.4 |
