# Supplementary material for: Duckweed Evolution: from Land back to Water
Source: Genomics Proteomics Bioinformatics. 2025 Aug 23;23(4):qzaf074. doi: 10.1093/gpbjnl/qzaf074 (PMC12707978; doi:10.1093/gpbjnl/qzaf074)
Supplement: qzaf074_Supplementary_Data [file qzaf074_supplementary_data.zip › Table_S32.docx]

Table S28 Summary of assessment of genome assembly with expressed sequence tags

| **Total** | **None** | **Short** | **Unique** | **Multi** |
| --- | --- | --- | --- | --- |
| 3794 | 21 | 53 | 3074 | 646 |
| Percent (%) | 0.6 | 1.4 | 81.0 | 17.0 |
