# Supplementary material for: Duckweed Evolution: from Land back to Water
Source: Genomics Proteomics Bioinformatics. 2025 Aug 23;23(4):qzaf074. doi: 10.1093/gpbjnl/qzaf074 (PMC12707978; doi:10.1093/gpbjnl/qzaf074)
Supplement: qzaf074_Supplementary_Data [file qzaf074_supplementary_data.zip › Table_S33.docx]

Table S33 Characteristics of RNA-sequencing data

| **Species** | **Reads length (bp)** | **Raw data (bp)** | **Clean data (bp)** | **Q20 (%)** |
| --- | --- | --- | --- | --- |
| *Spirodela polyrhiza* | 150 | 28,409,395,500 | 27,070,171,895 | 98.0 |
| *Landoltia punctata* | 150 | 28,529,510,400 | 27,023,351,344 | 98.1 |
| *Lemna minor* | 150 | 26,161,761,000 | 24,960,421,446 | 98.3 |
