# Supplementary material for: Duckweed Evolution: from Land back to Water
Source: Genomics Proteomics Bioinformatics. 2025 Aug 23;23(4):qzaf074. doi: 10.1093/gpbjnl/qzaf074 (PMC12707978; doi:10.1093/gpbjnl/qzaf074)
Supplement: qzaf074_Supplementary_Data [file qzaf074_supplementary_data.zip › Table_S34.docx]

Table S34 The results of clean reads alignment with rRNA

| **Species** | **All reads number** | **Mapped reads** | **Unmapped reads** |
| --- | --- | --- | --- |
| *Spirodela polyrhiza* | 184,608,218 | 68,173,988 (36.9%) | 116,434,230 (63.1%) |
| *Landoltia punctata* | 186,161,110 | 56,833,736 (30.5%) | 129,327,374 (69.5%) |
| *Lemna minor* | 170,702,602 | 50,181,076 (29.4%) | 120,521,526 (70.6%) |

*Note*: rRNA, ribosomal RNA.
