# Supplementary figures and images for: Duckweed Evolution: from Land back to Water
Source: Genomics Proteomics Bioinformatics. 2025 Aug 23;23(4):qzaf074. doi: 10.1093/gpbjnl/qzaf074 (PMC12707978; doi:10.1093/gpbjnl/qzaf074)

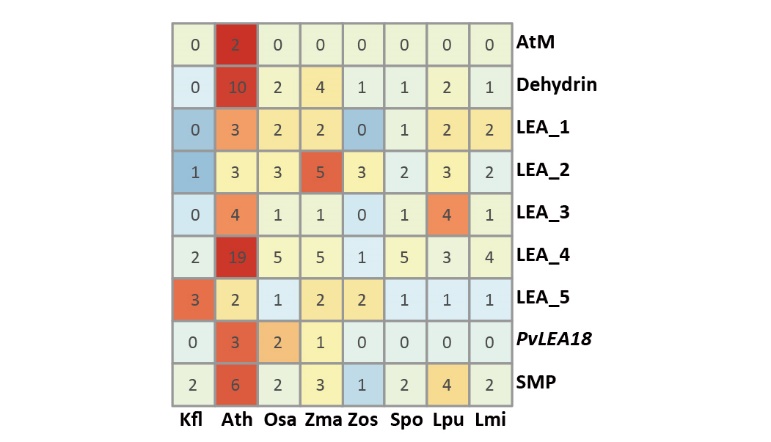

Supplement: qzaf074_Supplementary_Data [file qzaf074_supplementary_data.zip › Figure_S8.docx]

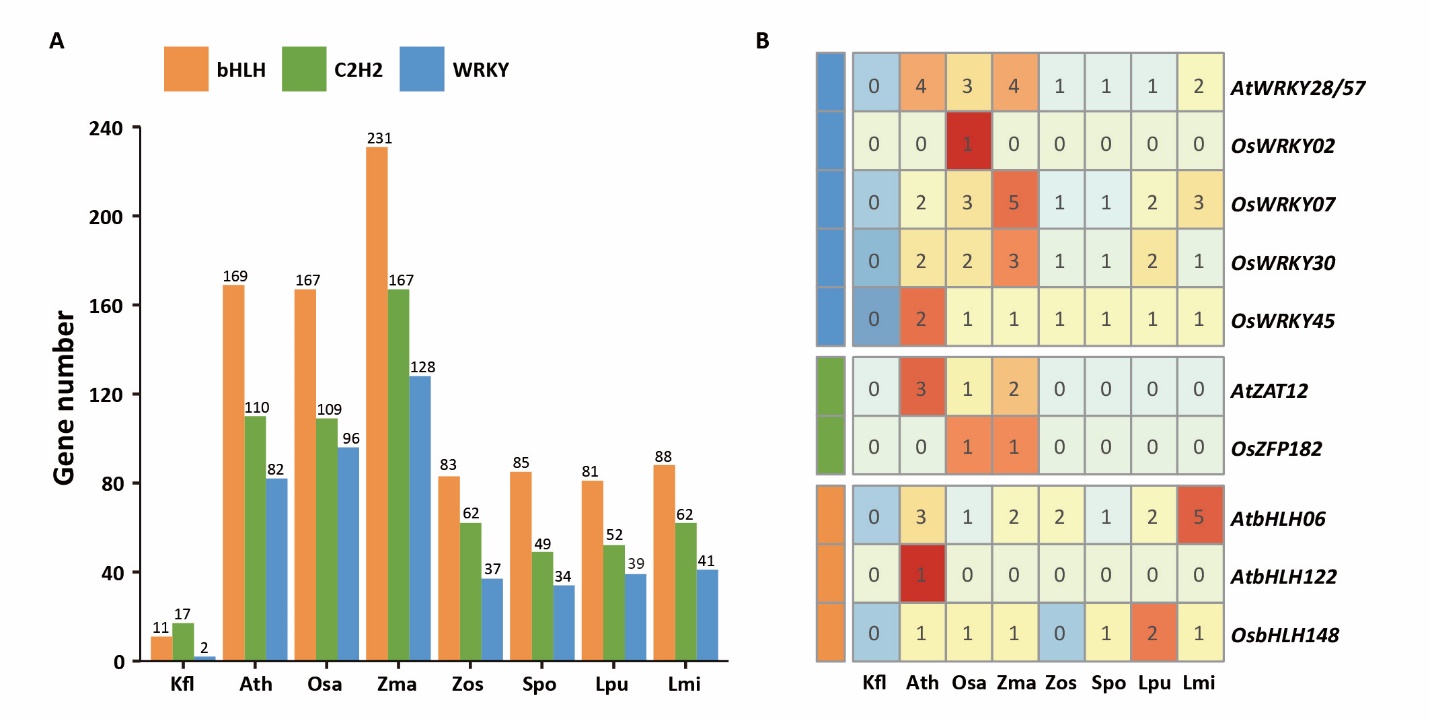

Supplement: qzaf074_Supplementary_Data [file qzaf074_supplementary_data.zip › Figure_S9.docx]

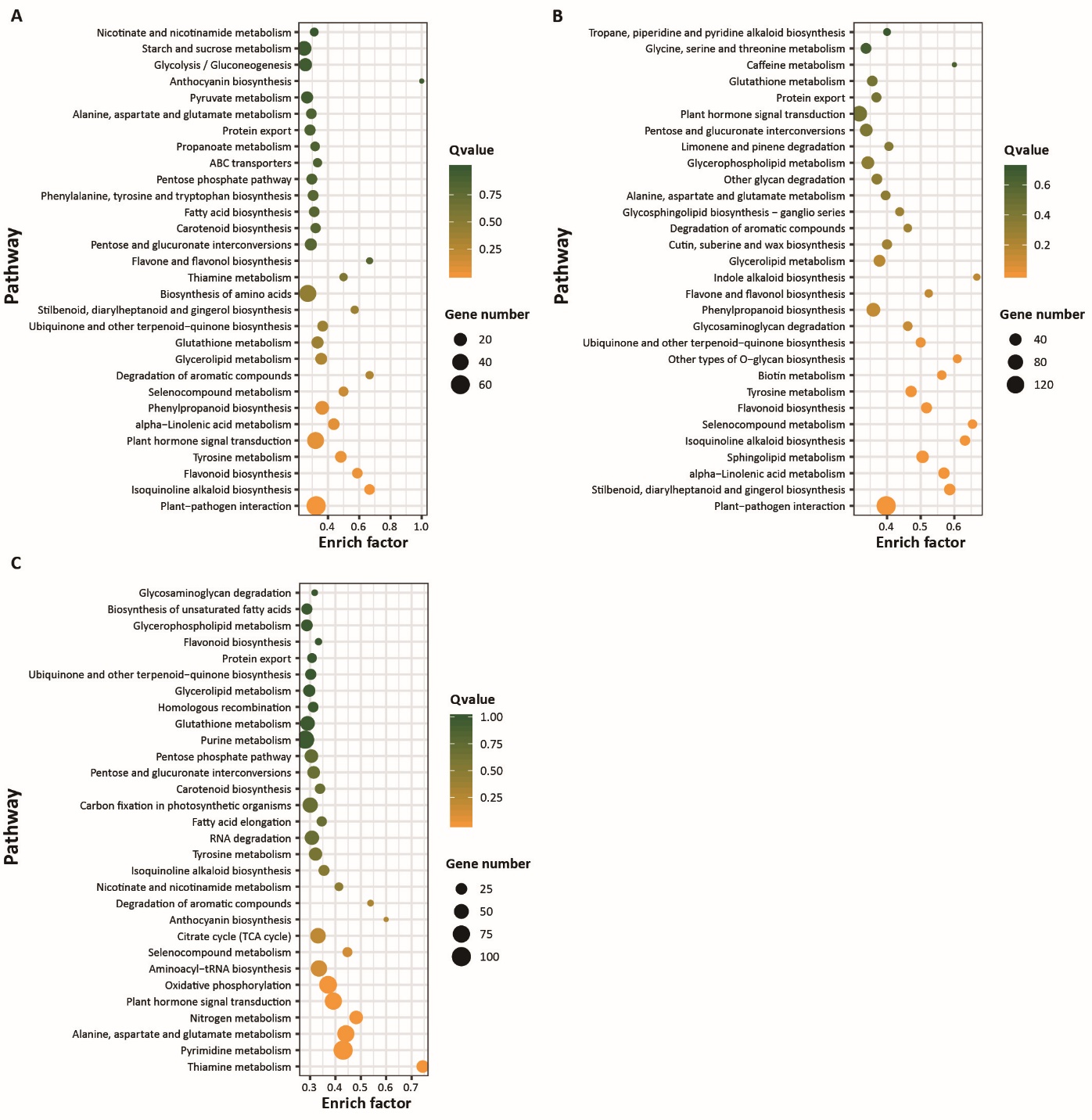

Supplement: qzaf074_Supplementary_Data [file qzaf074_supplementary_data.zip › Figure_S10.docx]

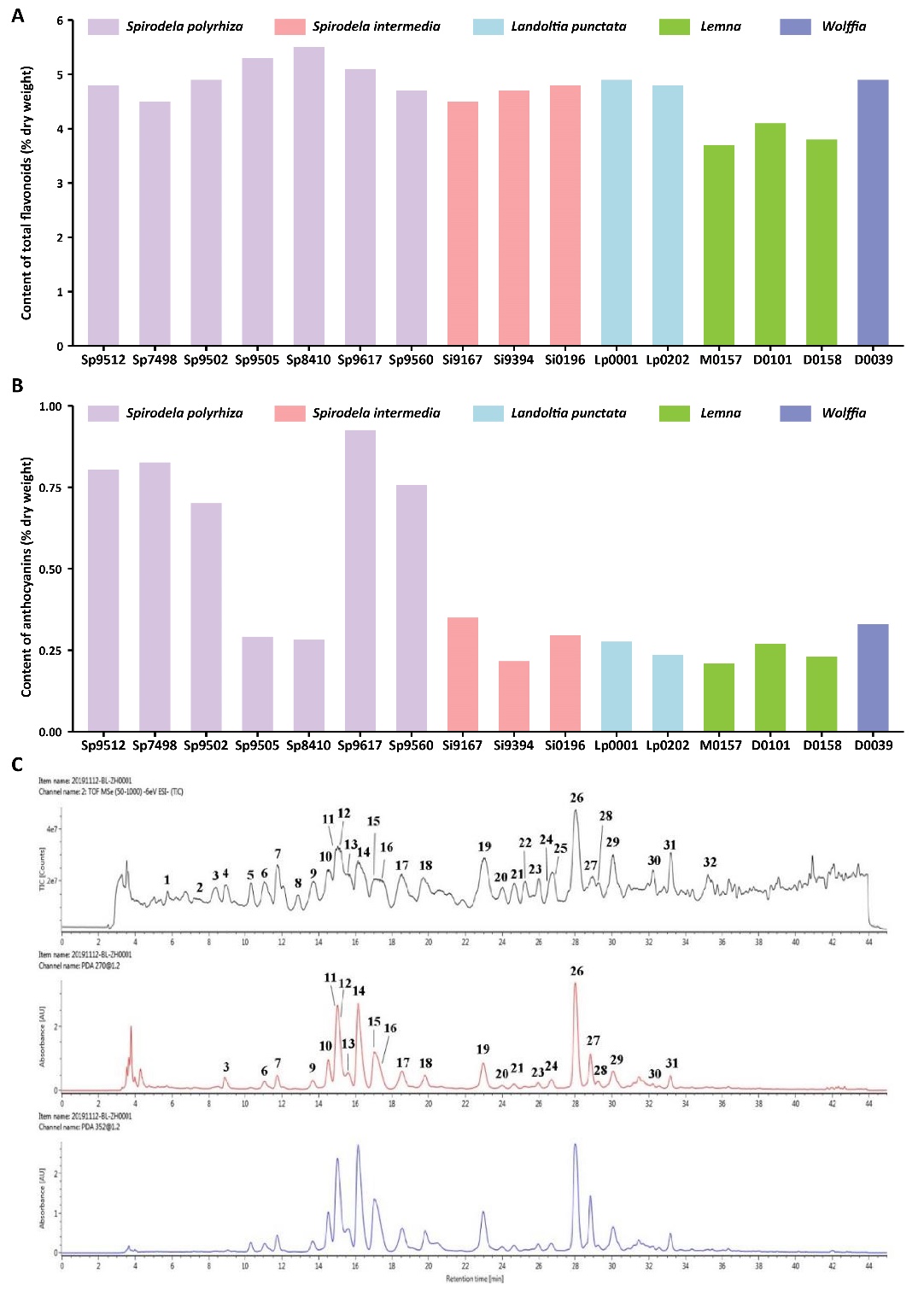

Supplement: qzaf074_Supplementary_Data [file qzaf074_supplementary_data.zip › Figure_S11.docx]

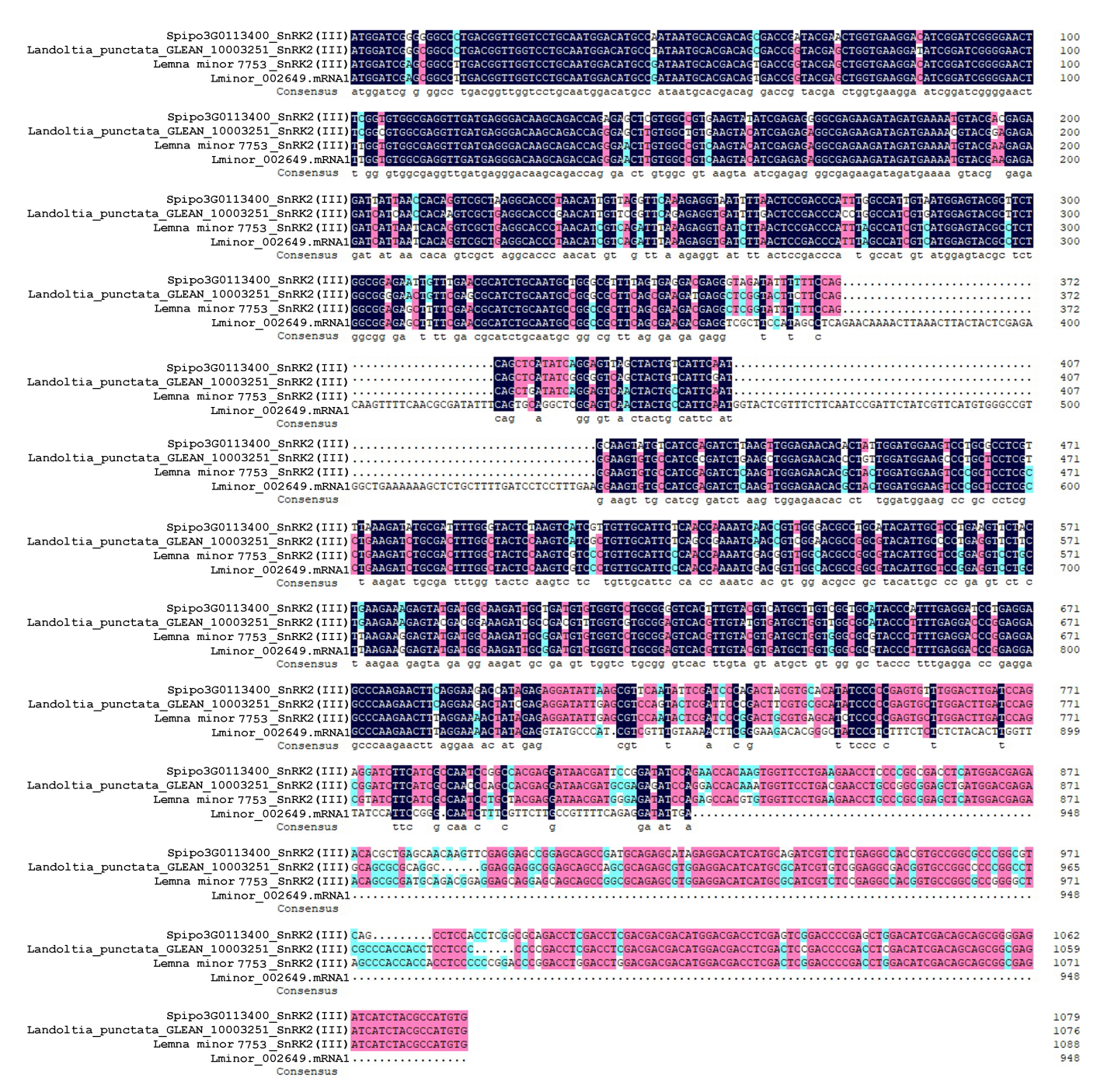

Supplement: qzaf074_Supplementary_Data [file qzaf074_supplementary_data.zip › Figure_S12.docx]

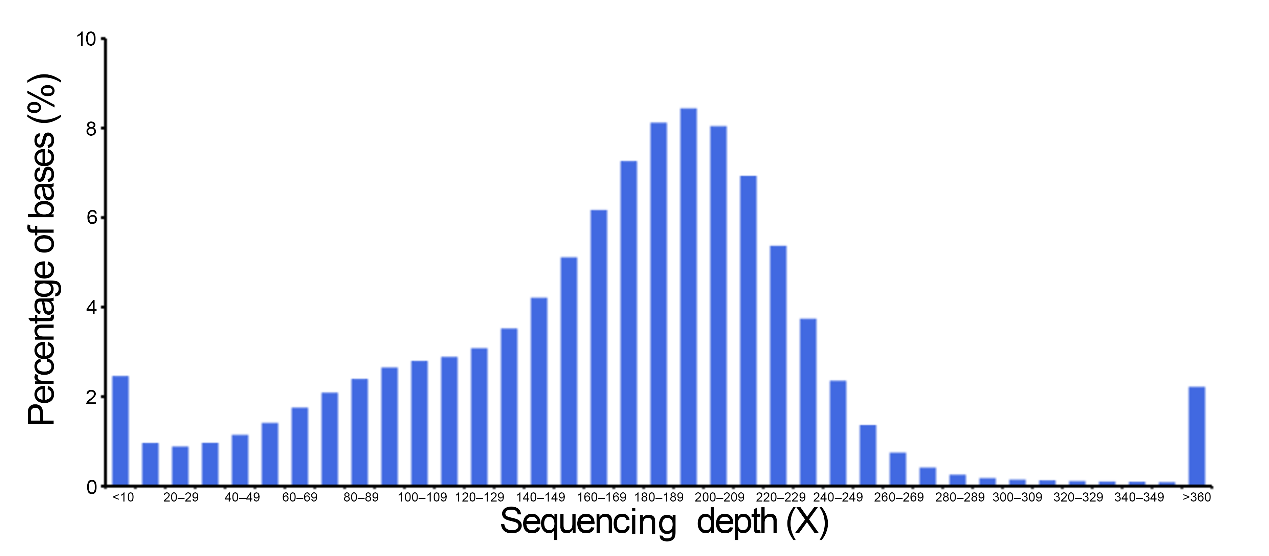

Supplement: qzaf074_Supplementary_Data [file qzaf074_supplementary_data.zip › Figure_S14.docx]

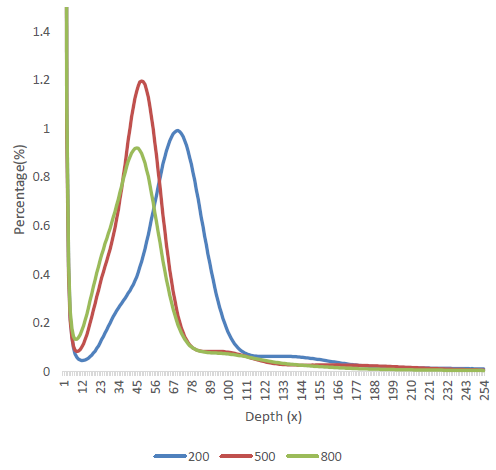

Supplement: qzaf074_Supplementary_Data [file qzaf074_supplementary_data.zip › Figure_S15.docx]

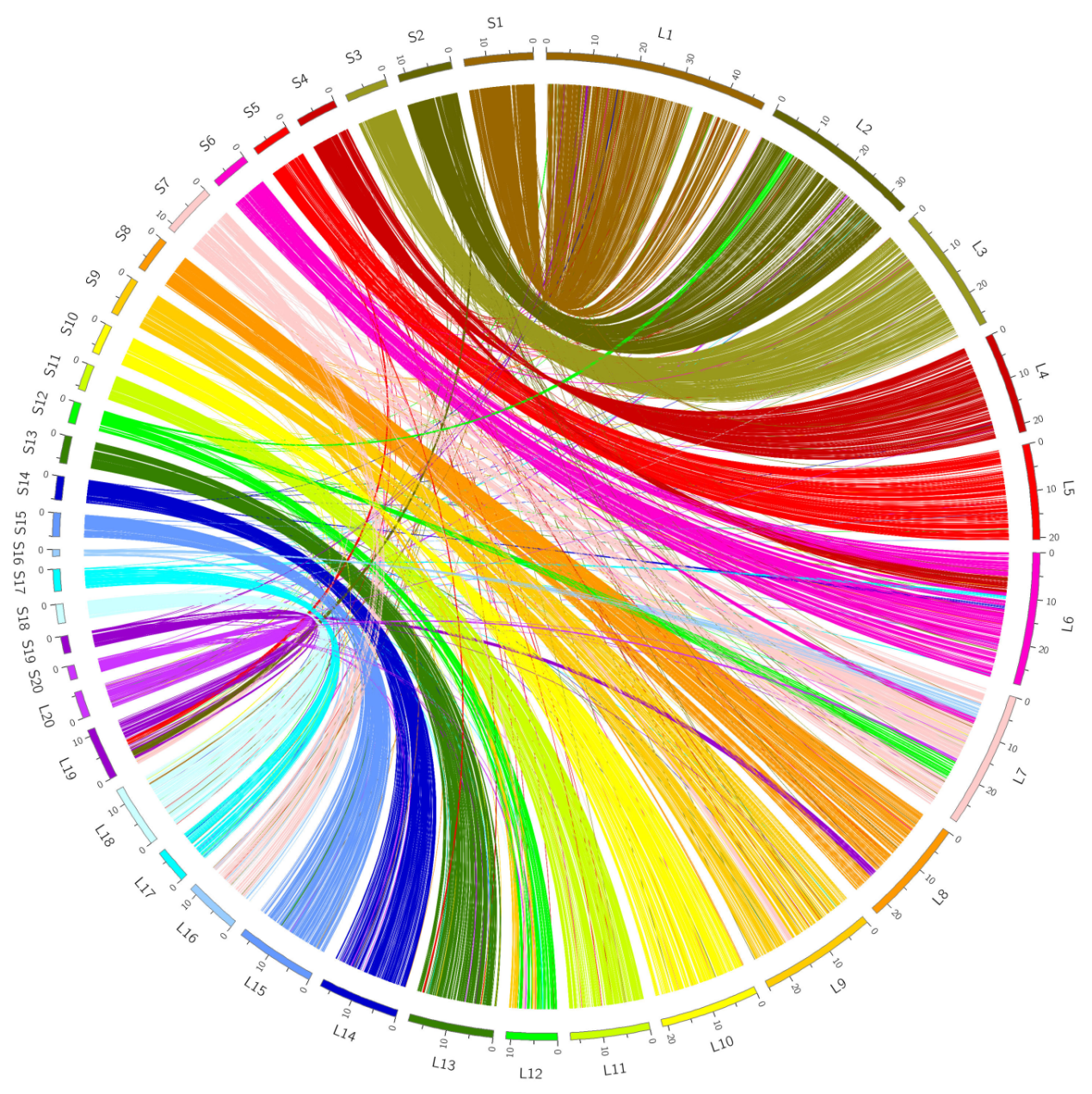

Supplement: qzaf074_Supplementary_Data [file qzaf074_supplementary_data.zip › Figure_S16.docx]

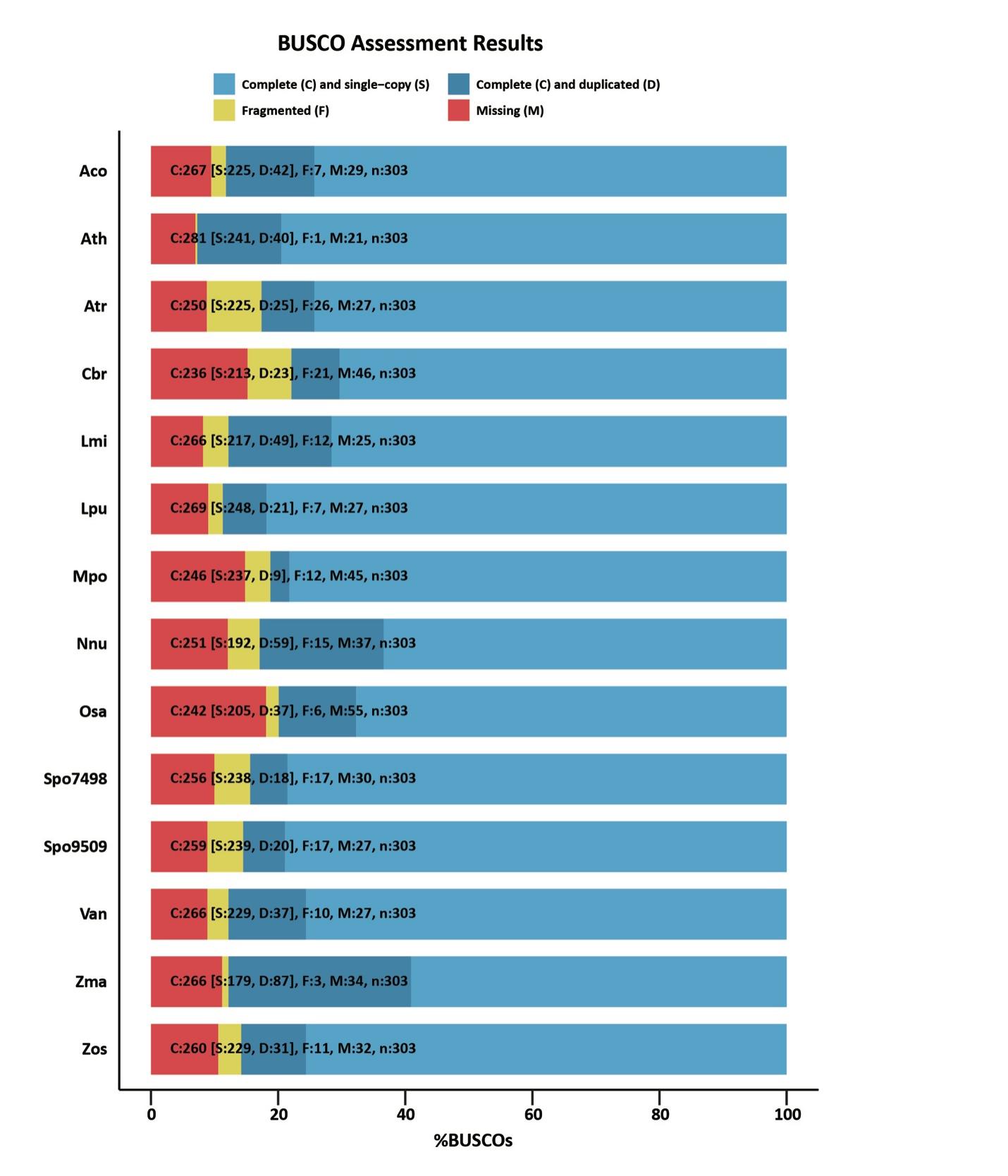

Supplement: qzaf074_Supplementary_Data [file qzaf074_supplementary_data.zip › Figure_S17.docx]

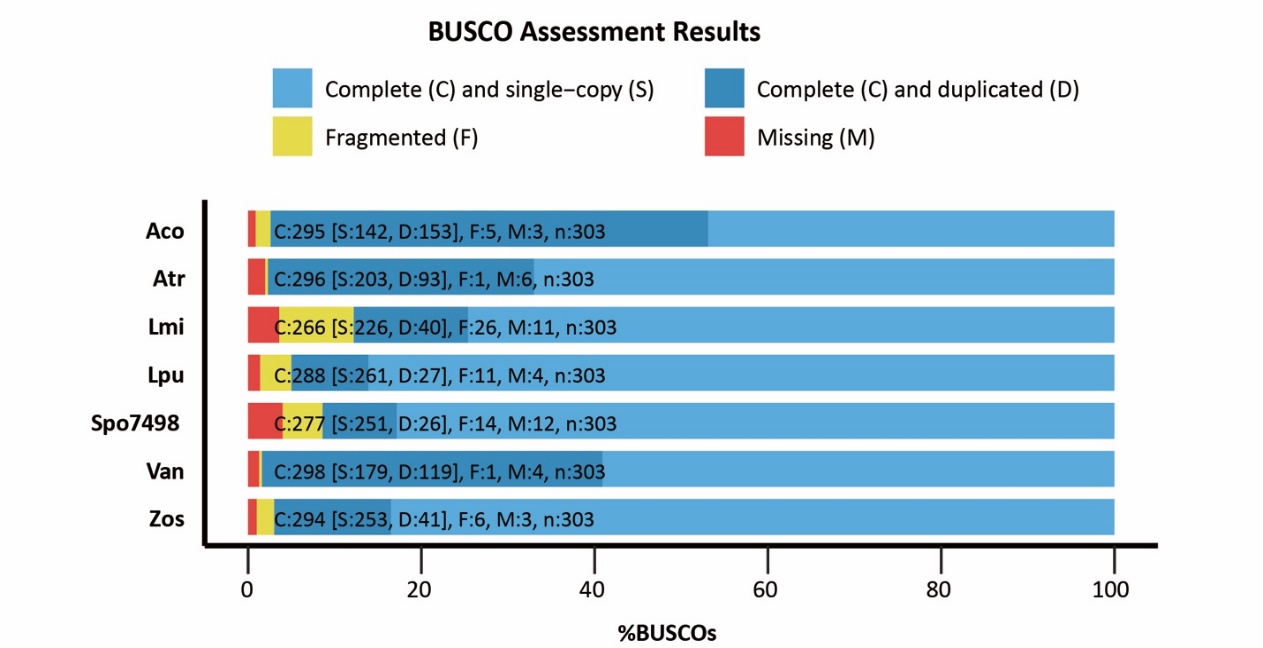

Supplement: qzaf074_Supplementary_Data [file qzaf074_supplementary_data.zip › Figure_S18.docx]

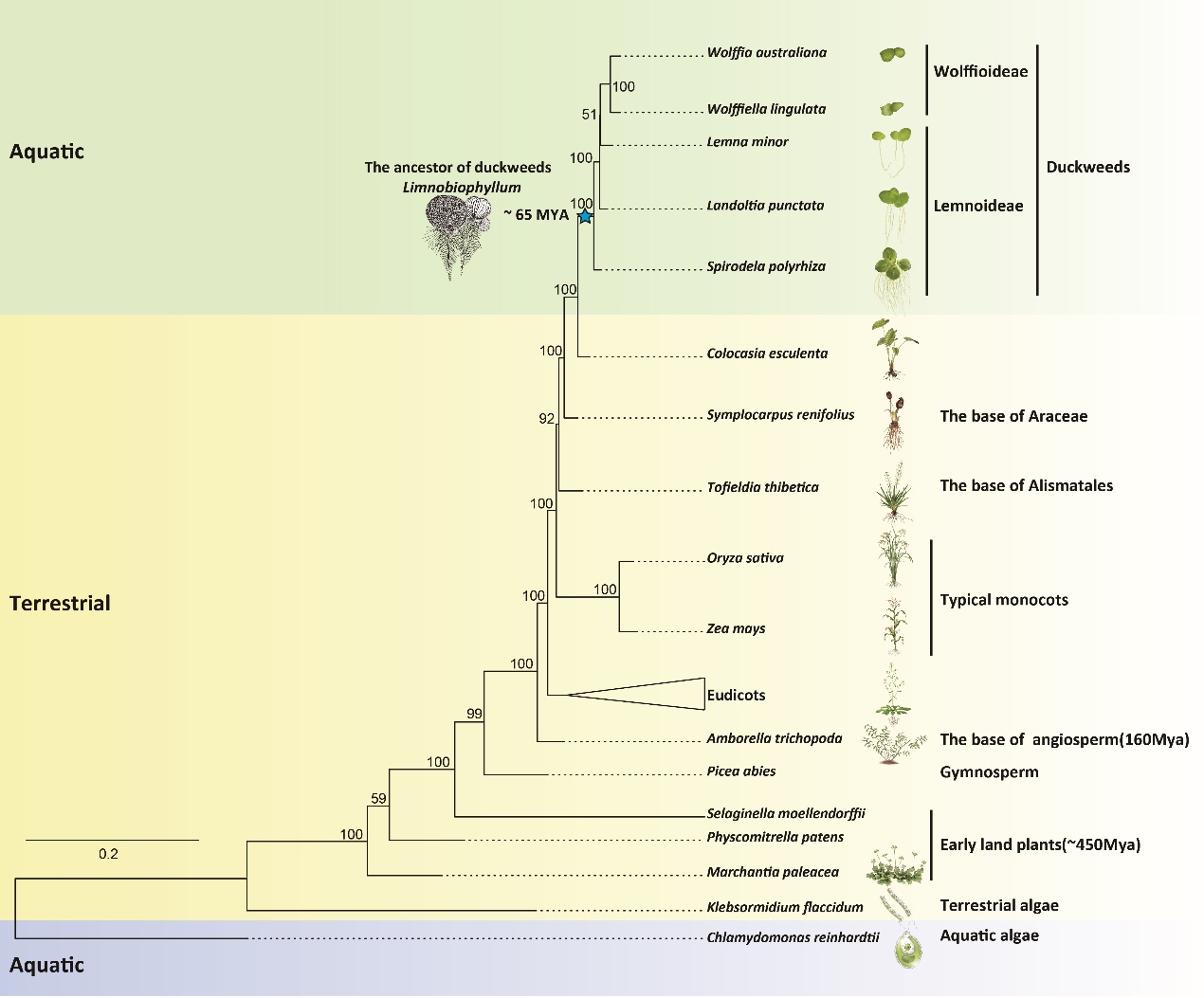

Supplement: qzaf074_Supplementary_Data [file qzaf074_supplementary_data.zip › Figure_S1.docx]

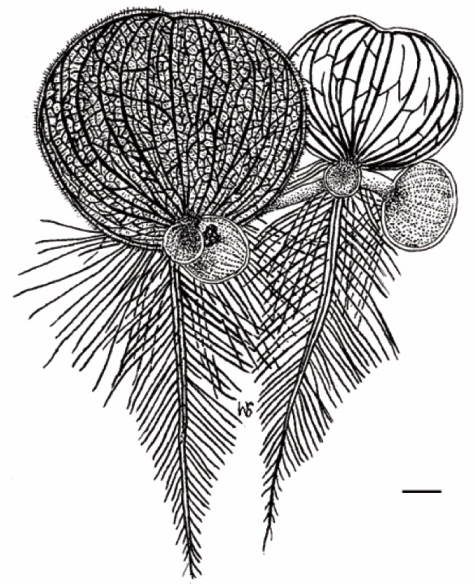

Supplement: qzaf074_Supplementary_Data [file qzaf074_supplementary_data.zip › Figure_S2.docx]

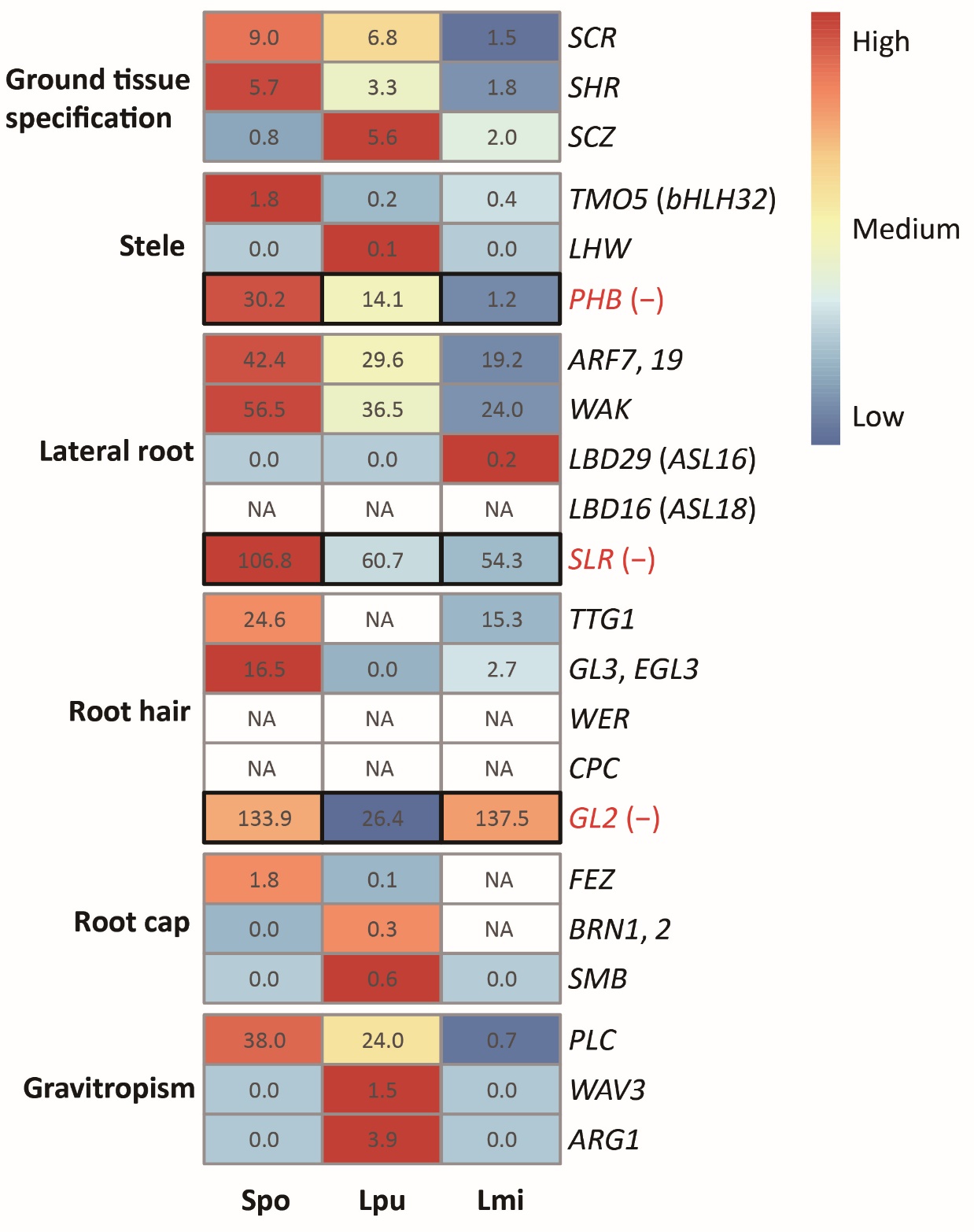

Supplement: qzaf074_Supplementary_Data [file qzaf074_supplementary_data.zip › Figure_S3.docx]

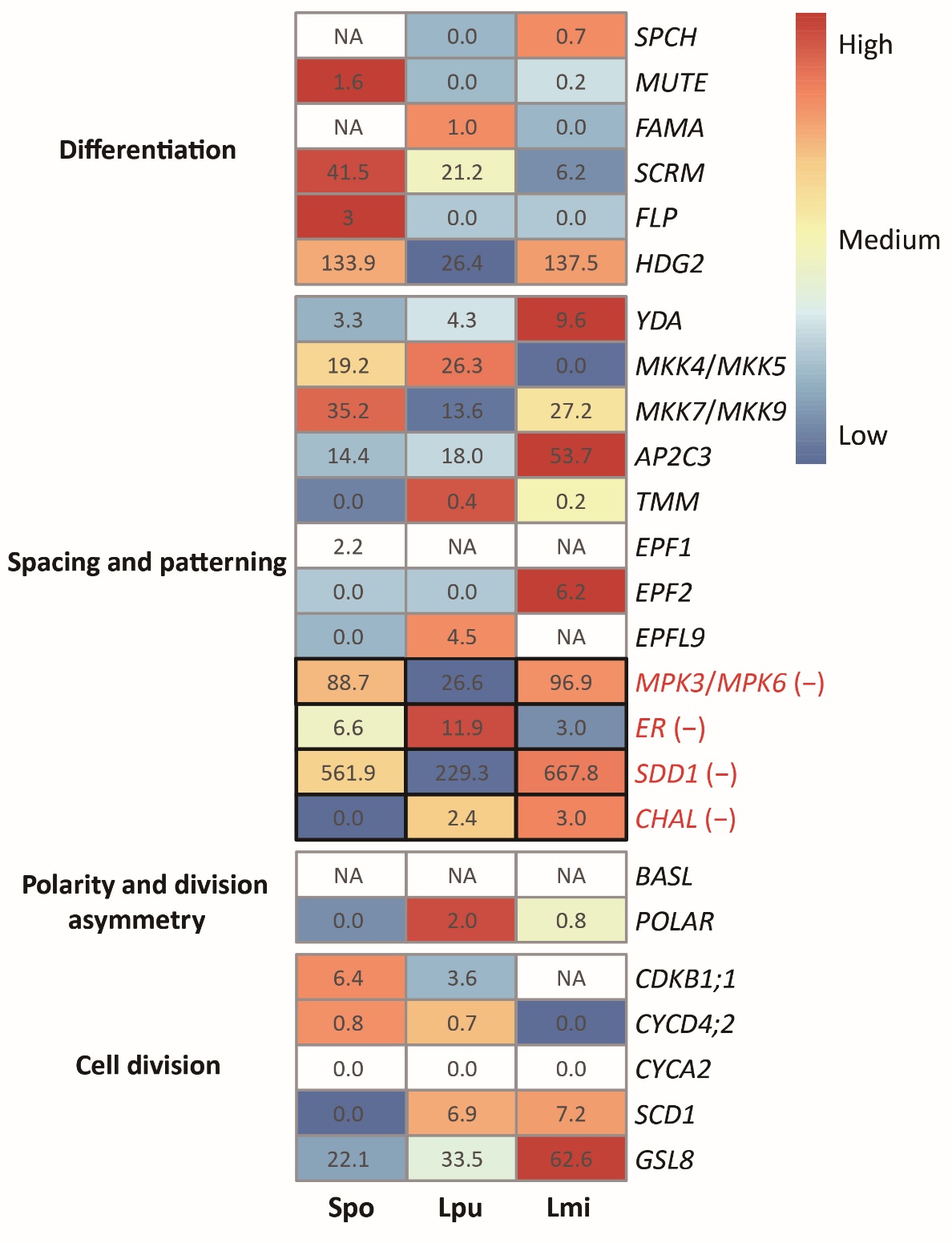

Supplement: qzaf074_Supplementary_Data [file qzaf074_supplementary_data.zip › Figure_S4.docx]

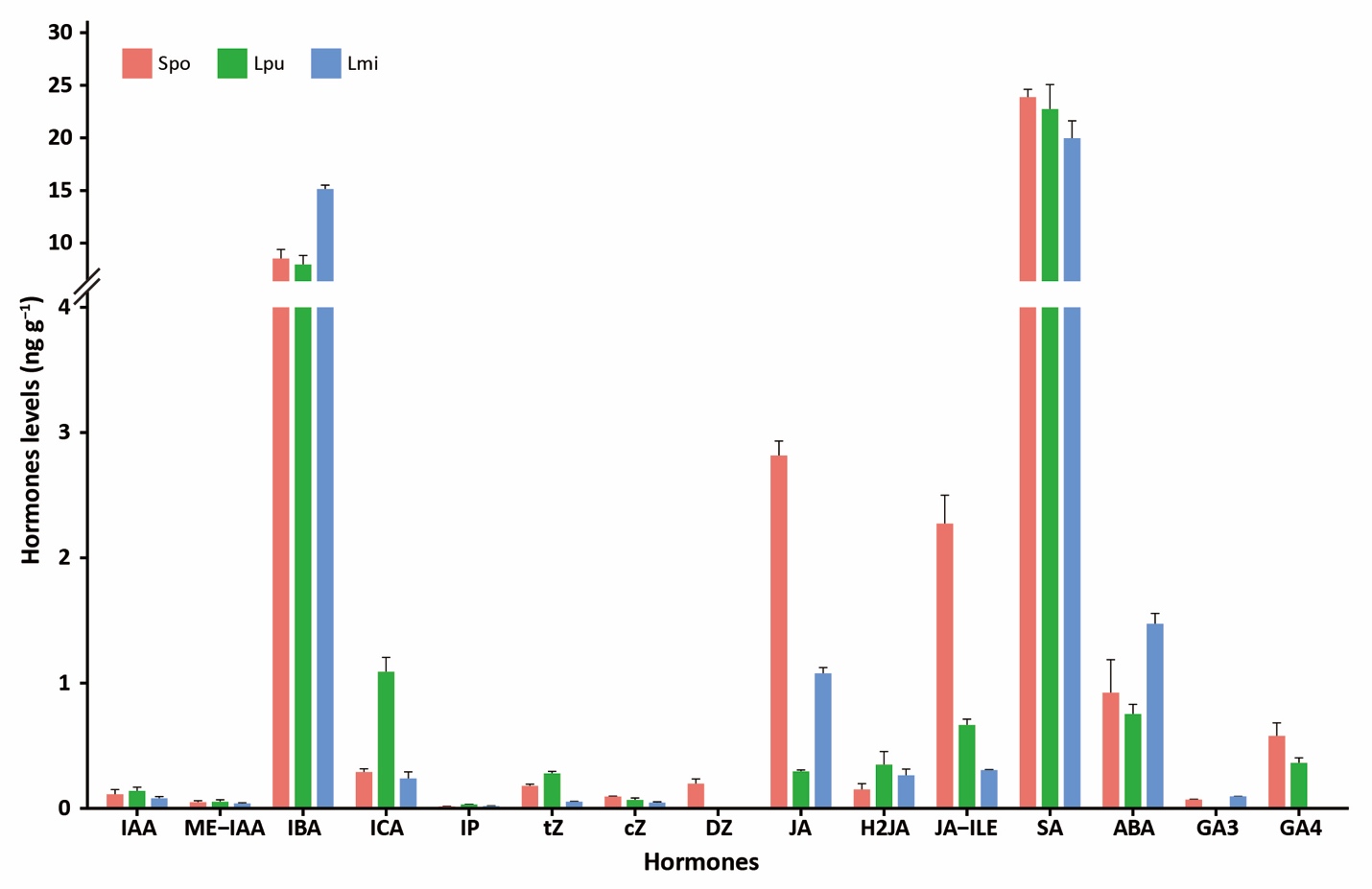

Supplement: qzaf074_Supplementary_Data [file qzaf074_supplementary_data.zip › Figure_S5.docx]

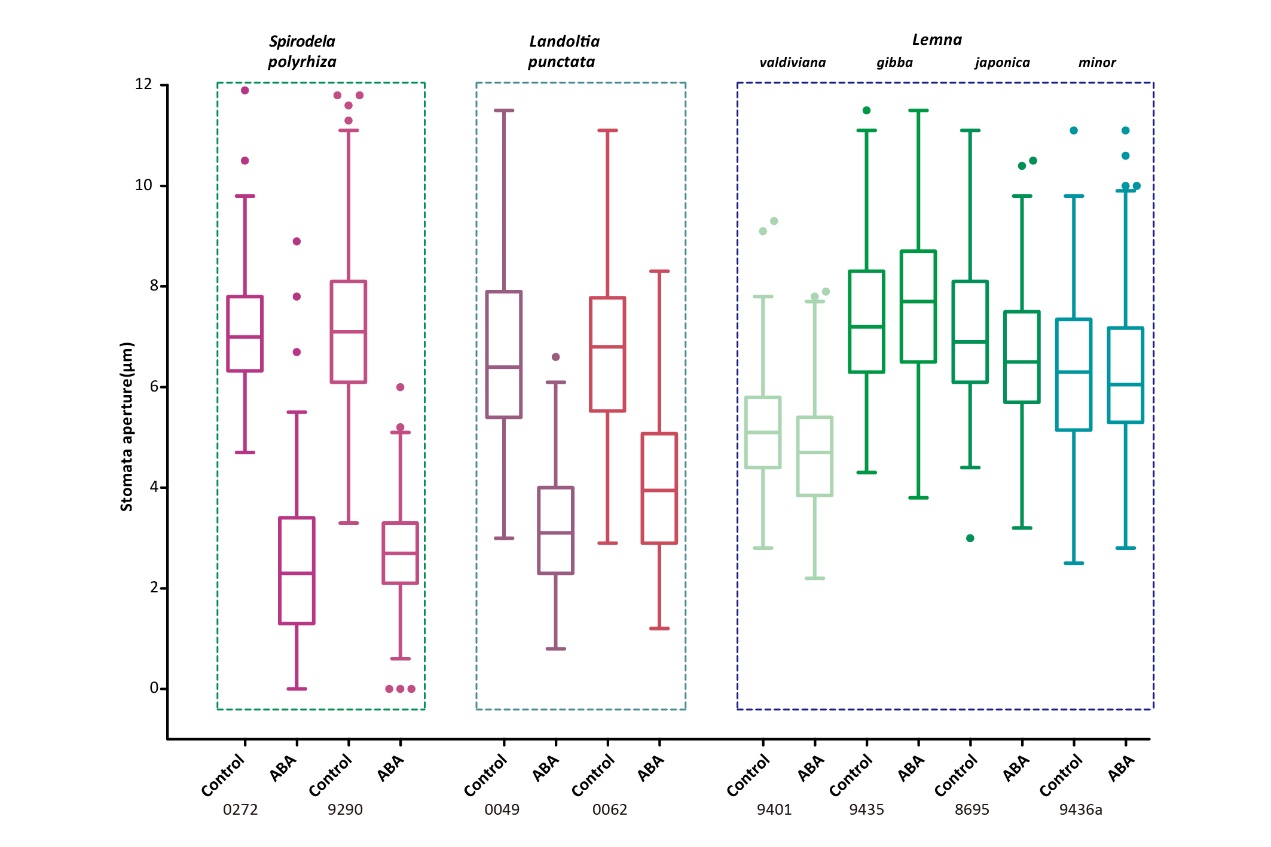

Supplement: qzaf074_Supplementary_Data [file qzaf074_supplementary_data.zip › Figure_S6.docx]

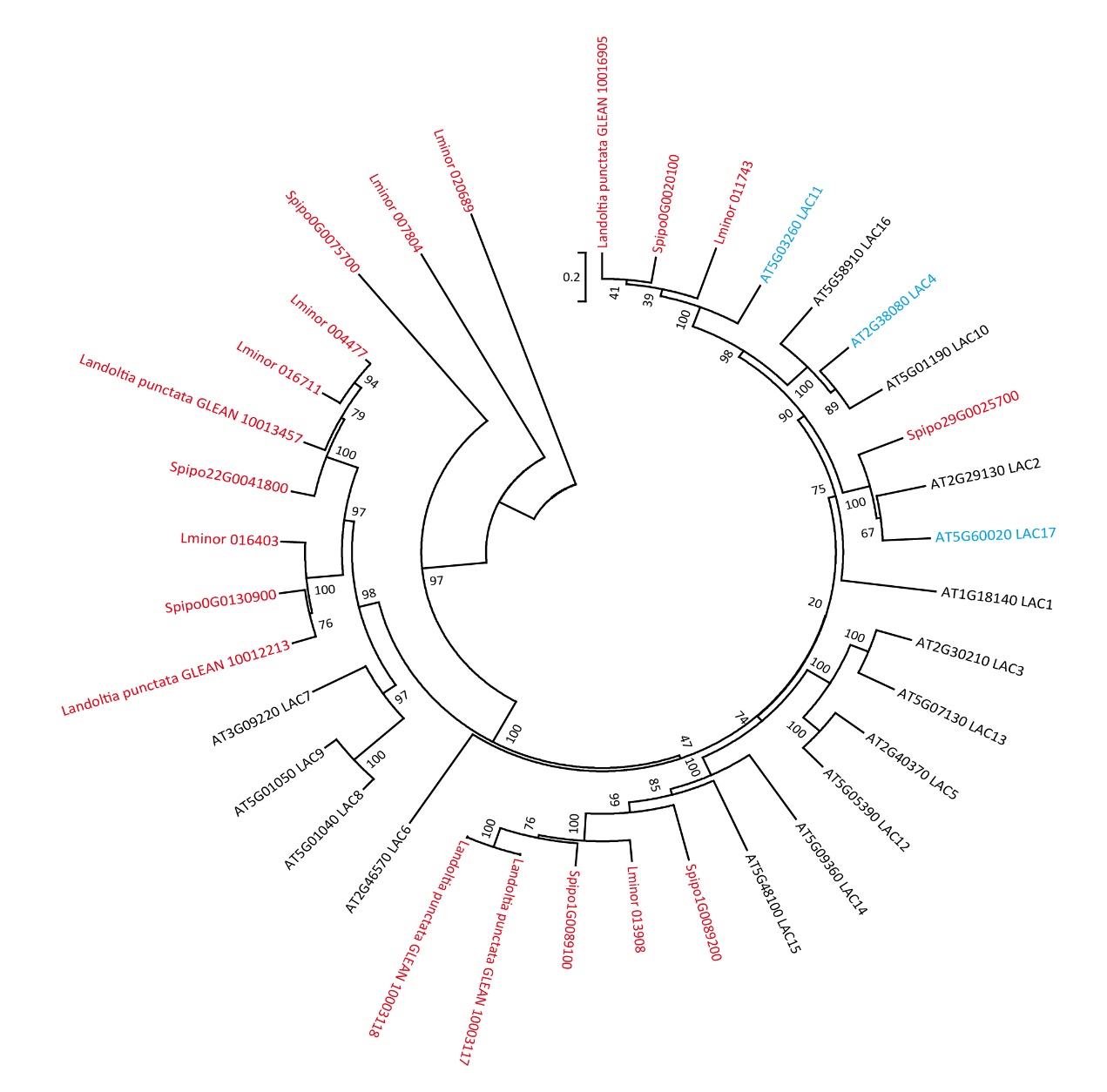

Supplement: qzaf074_Supplementary_Data [file qzaf074_supplementary_data.zip › Figure_S7.docx]
